# Metallothionein 2A Expression in Cancer-Associated Fibroblasts and Cancer Cells Promotes Esophageal Squamous Cell Carcinoma Progression

**DOI:** 10.3390/cancers13184552

**Published:** 2021-09-10

**Authors:** Masaki Shimizu, Yu-ichiro Koma, Hiroki Sakamoto, Shuichi Tsukamoto, Yu Kitamura, Satoshi Urakami, Kohei Tanigawa, Takayuki Kodama, Nobuhide Higashino, Mari Nishio, Manabu Shigeoka, Yoshihiro Kakeji, Hiroshi Yokozaki

**Affiliations:** 1 Division of Pathology, Department of Pathology, Kobe University Graduate School of Medicine, Kobe 650-0017, Japan; smasaki@med.kobe-u.ac.jp (M.S.); hiroki3_21@yahoo.co.jp (H.S.); stsuka@med.kobe-u.ac.jp (S.T.); y.kitamura-0916@people.kobe-u.ac.jp (Y.K.); urasato@med.kobe-u.ac.jp (S.U.); 188m863m@gsuite.kobe-u.ac.jp (K.T.); takodama@med.kobe-u.ac.jp (T.K.); higasinobu1108@msn.com (N.H.); marin@med.kobe-u.ac.jp (M.N.); mshige@med.kobe-u.ac.jp (M.S.); hyoko@med.kobe-u.ac.jp (H.Y.); 2 Division of Gastro-intestinal Surgery, Department of Surgery, Kobe University Graduate School of Medicine, Kobe 650-0017, Japan; kakeji@med.kobe-u.ac.jp; 3 Division of Gastroenterology, Department of Internal Medicine, Kobe University Graduate School of Medicine, Kobe 650-0017, Japan

**Keywords:** cancer-associated fibroblasts, esophageal squamous cell carcinoma, MT2A, IGFBP2

## Abstract

**Simple Summary:**

Cancer-associated fibroblasts (CAFs) are tumor promoters in various cancers. We previously reported a correlation between the high expression of the CAF marker fibroblast activation protein and poor prognosis of esophageal squamous cell carcinoma (ESCC). We also found that metallothionein 2A (MT2A) is highly expressed in CAF-like cells that we established. In the current study, we explored the role of MT2A in ESCC progression. MT2A expression in the CAF-like cells induced expression and secretion of insulin-like growth factor binding protein 2 (IGFBP2), which promoted the migration and invasiveness of ESCC cells through the NFκB, Akt, and Erk signaling pathways. Furthermore, MT2A was involved in ESCC cell growth, migration, and invasiveness. Moreover, high expression of MT2A in the cancer tissue correlated with poor prognosis of ESCC patients. Briefly, we demonstrate that MT2A and IGFBP2 are potential novel therapeutic targets in ESCC.

**Abstract:**

Esophageal cancer has the sixth highest mortality rate worldwide. Cancer-associated fibroblasts (CAFs) are involved in the progression of various cancers. Previously, we demonstrated an association between high expression of the CAF marker, fibroblast activation protein, and poor prognosis of esophageal squamous cell carcinoma (ESCC). We also established CAF-like cells by indirect co-culture of bone marrow-derived mesenchymal stem cells with ESCC cell lines and found metallothionein 2A (MT2A) to be highly expressed in them. Here, to explore the function of MT2A in CAFs, we silenced *MT2A* in the CAF-like cells and ESCC cell lines using small interfering RNA. *MT2A* knockdown in the CAF-like cells suppressed expression and secretion of insulin-like growth factor binding protein 2 (IGFBP2); recombinant IGFBP2 promoted migration and invasiveness of ESCC cells via NFκB, Akt, and Erk signaling pathways. Furthermore, *MT2A* knockdown in the ESCC cell lines inhibited their growth, migration, and invasiveness. Immunohistochemistry demonstrated that high MT2A expression in the cancer stroma and cancer nest of ESCC tissues correlated with poor prognosis of ESCC patients. Hence, we report that MT2A in CAFs and cancer cells contributes to ESCC progression. MT2A and IGFBP2 are potential novel therapeutic targets in ESCC.

## 1. Introduction

Cancer is a significant global health problem and the second leading cause of death in the world [1]. Among all cancers, esophageal cancer is the seventh most common globally, with over 600,000 new cases in 2020 [1]. Esophageal cancer also has the sixth highest mortality rate in the world, having caused over 500,000 deaths per year (accounting for 1 in every 18 cancer-related deaths in 2020) [1]. Esophageal cancer can be classified into two major histological subtypes: esophageal squamous cell carcinoma (ESCC) and esophageal adenocarcinoma (EAC) [2,3]. ESCC is the most common type in East Asia (including Japan), Central Asia, East Africa, and South Africa, while EAC is the predominant type in North America and Europe [4,5]. Globally, ESCC accounts for about 90% of esophageal cancer cases, and its incidence is approximately seven times that of EAC [6]. Therefore, studying the mechanisms of ESCC development and progression is essential for global health.

In the early stages of ESCC, as the carcinoma is limited to the mucosa, it can be entirely resected endoscopically, and patients have a good 5-year survival rate of over 90% [7,8]. However, at later stages, when the ESCC invades the submucosal layer, patients have a poor 5-year survival rate of approximately 70%, which can even be as low as 43.4–65.8% in the most invasive cases where the tumor submucosal infiltration is extensive [8]. Owing to histological and anatomical factors, advanced ESCC is refractory, with poor prognosis compared with early ESCC. Histologically, the esophageal submucosa is rich in reticulated lymphatic vessels [9]. Therefore, as the tumor invades deeper into the submucosa, the frequency of lymph node metastases increases [10]. Additionally, as the ESCC tumors extend beyond the adventitia, the anatomical proximity of the esophagus to essential organ parts, such as the aorta, trachea, pulmonary artery, pericardium, and pleura, allows greater infiltration of the tumors into adjacent organs [11]. In such cases, radical surgical resection becomes impossible, making it crucial to further elucidate the mechanisms of ESCC invasion.

The tumor microenvironment (TME) consists of tumor cells and non-tumor cells including fibroblasts, endothelial cells, and immune cells, such as macrophages and lymphocytes [12]. The non-tumor cells are essential for tumor progression, and hence are important therapeutic targets. For example, tumor cells achieve immune escape through the binding of programmed death ligand-1 (PD-L1) on their surface to programmed death-1 (PD1) expressed on T-lymphocytes, which suppresses activation of the latter [13,14]. In fact, antibodies targeting PD-L1 or PD1 have been used in clinical practice for the treatment of esophageal cancer as well as other malignant tumors such as melanoma, non-small cell lung cancer, renal cell carcinoma, Hodgkin lymphoma, head and neck cancer, and gastric cancer. Additionally, cancer-associated fibroblasts (CAFs) and tumor-associated macrophages (TAMs), major cell populations in the TME, play important roles in tumor development [15]. Accumulating evidence indicates that the molecular mechanisms underlying CAFs and TAMs hold the key to tumor development in many cancers, including ESCCs, and have hence attracted much attention as novel therapeutic targets [15].

Previously, we reported that high expression of the CAF marker, fibroblast activation protein (FAP), is associated with poor prognosis of ESCC. Furthermore, CAF-like cells, which we established by indirect co-culture of human bone marrow-derived mesenchymal stem cells (MSCs, one of the origins of CAFs) with ESCC cell lines, were found to play a tumor-promoting role in the ESCC microenvironment through IL-6 and CCL2 using cytokine array analysis. Additionally, the CAF-like cells promoted migration and invasion by ESCC cells and induced migration and TAM-like polarization of macrophages [16]. Our cDNA microarray analysis suggested that plasminogen activator inhibitor-1 (PAI-1) promoted the CAF-like cell-induced migration and invasion by ESCC cells and macrophages through the activation of Akt and Erk signaling via LDL receptor-related protein 1 (LRP1) [17]. The CAF-like cells also highly expressed IL-6 and CCL2 by cDNA microarray analysis [17]. The role of IL-6 and CCL-2 in tumor progression was also demonstrated by Qin et al. and Tsuyada et al., respectively [18,19]. IL-6 secreted by primary CAFs isolated from the tumor tissues of head and neck cancer (HNC) patients promoted HNC progression through the osteopontin-nuclear factor kappa B (NFκB) signaling pathway [18], whereas CCL2 produced by breast cancer primary CAFs promoted cancer advancement by regulating cancer stem cells through NOTCH activation [19]. Tsuyada et al. also reported that paracrine signaling by breast cancer cells induced CCL2 secretion from primary CAFs through STAT3 activation [19]. Moreover, another study found that treatment with cisplatin induced PAI-1 secretion by primary CAFs, which subsequently promoted ESCC progression [20]. These reports suggested that IL-6, CCL2, and PAI-1 produced by the CAF-like cells were also expressed in primary CAFs isolated from tumor tissue. Hence, CAF-like cells established in our previous research are similar to primary CAFs obtained from cancer tissues and can be used to explore mechanisms underlying the tumor-promoting roles of CAFs.

Our previous cDNA microarray analysis revealed that the metallothionein 2A (*MT2A*) gene was the most upregulated in our CAF-like cells compared with that in the MSCs. In this present study, we followed up on our research and investigated the role of MT2A in ESCC progression using the CAF-like cells and ESCC cell lines and determined whether it could be used as a potential therapeutic target in the disease.

## 2. Materials and Methods

### 2.1. Cell Lines and Cell Culture

ESCC cell lines, TE-8, TE-9, TE-10, TE-11, and TE-15, obtained from the RIKEN BioResource Center (Tsukuba, Japan), were maintained in RPMI-1640 medium (Wako, Osaka, Japan) supplemented with 10% fetal bovine serum (FBS; Sigma-Aldrich, St. Louis, MO, USA) and 1% antibiotic-antimycotic (Invitrogen, Carlsbad, CA, USA). Human bone marrow-derived mesenchymal stem cells (MSCs) were purchased from the American Type Culture Collection (ATCC^®^ PCS-500-012^TM^; Manassas, VA, USA). MSCs were maintained in low-glucose DMEM (Wako) supplemented with 10% FBS and 1% antibiotic-antimycotic.

### 2.2. Preparation of CAF-Like Cells

The CAF-like cells were established by a previously described method [16]. In brief, 5 × 10^4^ MSCs were seeded to the bottom chamber of 6-well plates and co-cultured with 1.5 × 10^5^ ESCC cells in the upper chamber (0.4 μm pore size filter; BD Falcon, Lincoln Park, NY, USA) for 7 days. The medium was changed 4 days after seeding. MSCs seeded in the same manner without co-culture were used as a control. After confirming the higher expression levels of the CAF marker, FAP, in MSCs co-cultured with ESCC cells than in the monocultured MSCs (Figure S1 and Figure S8I), we labeled the MSC-derived CAF-like cells obtained from the co-culture of TE-8, TE-9, and TE-15 cells as CAF8, CAF9, and CAF15, respectively.

### 2.3. Reverse Transcription Polymerase Chain Reaction (RT-PCR) and Quantitative Real-Time-PCR (qRT-PCR)

We extracted total RNA from the cells using RNeasy Mini Kit (Qiagen, Hilden, Germany). The expression of *MT2A* and the internal control gene, glyceraldehyde-3-phosphate dehydrogenase (*GAPDH*), was evaluated using RT-PCR. The sequences of the primers used were: *GAPDH*, 5′-ACCACAGTCCATGCCATCAC-3′ (forward) and 5′-TCCACCCTGTTGCTGTA-3′ (reverse); *MT2A*, 5′-AACCTGTCCCGACTCTAGC-3′ (forward) and 5′-GGAATATAGCAAACGGTCACG-3′ (reverse); *CDH1*, 5′-ACAGCACGTACACAGACCCTA-3′ (forward) and 5′-GCAGAAGTGTCCCTGTCCCAG-3′ (reverse). RT-PCR products were separated by electrophoresis in agarose gel (2%).

qRT-PCR was performed using the following probes: *FAP* (Hs00990806_m1), *MT2A* (Hs02379661_g1), *IGFBP2* (Hs0140719_m1), and *ACTB* (Hs01060665_g1) (Applied Biosystems, Foster City, CA, USA) on the ABI StepOnePlus Real-time PCR system (Applied Biosystems) using TaqMan^®^ Gene Expression Master Mix (Applied Biosystems). Target gene expression in the respective samples was normalized to the levels of the internal control *ACTB*.

### 2.4. Western Blotting

Cell lysates were prepared using RIPA Lysis and Extraction Buffer (Thermo Fisher Scientific, Waltham, MA, USA) containing 1% Protease Inhibitor and 1% Phosphatase Inhibitor Cocktail (Sigma-Aldrich). The lysates were resolved on 5–20% sodium dodecyl sulfate-polyacrylamide gels, and the resolved protein bands were transferred to a PVDF membrane using an iBlot^®^2 (Invitrogen). After blocking with 5% skim milk, the membrane was incubated with the primary antibodies and then with the corresponding secondary antibodies. Protein bands were detected using ImmunoStar Reagents (Wako).

The primary antibodies used were as follows. The sheep FAP antibody (AF3715) was obtained from R&D Systems (Minneapolis, MN, USA) and the mouse MT2A antibody (SAB1402848) from Sigma-Aldrich. The rest were all obtained from Cell Signaling Technology (Beverly, MA, USA): rabbit IGFBP2 (#3922), rabbit phosphorylated Akt (Ser473, #4060), rabbit phosphorylated Akt (Thr308, #2965), rabbit total Akt (#9272), rabbit phosphorylated Erk1/2 (Thr202/Tyr204, #9101), rabbit total Erk1/2 (#9102), rabbit phosphorylated NFκB (#3033), rabbit total NFκB (#8242), rabbit E-cadherin (#3195), rabbit β-catenin (#8480), rabbit phosphorylated β-catenin (Ser33/Ser37/Thr41, #9561 and Thr41/Ser45, #9565), and rabbit β-actin (#4970) antibodies. The secondary antibodies used were horseradish peroxidase (HRP)-conjugated donkey anti-rabbit IgG (NA934V; Cytiva, Chicago, IL, USA), sheep anti-mouse IgG (NA931V; Cytiva), and donkey anti-sheep IgG (ab6900; Abcam, Cambridge, UK).

### 2.5. Small Interfering (si)RNA in CAF-Like Cells and ESCC Cell Lines

CAF-like cells or ESCC cell lines were transfected with siRNA (20 pmol) targeting human *MT2A* (siMT2A, sc-93491; Santa Cruz Biotechnology, Dallas, TX, USA) or human *FAP* (siFAP, SASI_Hs02_00337654; Sigma-Aldrich) using Lipofectamine RNAiMAX (Invitrogen). MISSION^®^ siRNA Universal Negative Control #1 (siNC; Sigma-Aldrich) was used as the negative control.

### 2.6. Enzyme-Linked Immunosorbent Assay (ELISA)

The medium was changed in both the induced CAF-like cells and the control MSCs to fresh DMEM. The conditioned media (CM) were collected after 48 h and analyzed using the Human IGFBP-2 Quantikine ELISA Kit (R&D Systems) according to the manufacturer’s instructions. The optical density of each well was read using a microplate reader (Infinite 200 PRO; Tecan, Mannedorf, Switzerland) at 450 and 570 nm wavelengths, and the concentration of each cytokine was calculated from the measured absorbance using a standard curve.

### 2.7. Antibody Array

After the induction of monocultured MSCs (monoMSC), co-culture of MSCs with TE-15 (i.e., CAF15), and transfection of CAF15 with siNC (CAF15 siNC) or siMT2A (CAF15 siMT2A) as described above, the medium was changed to fresh DMEM. After 48 h, the CM was collected and analyzed using a Proteome Profiler Human XL Cytokine Array Kit (R&D Systems) according to the manufacturer’s instructions.

### 2.8. Cell Survival and Growth Assay

Cells (1 × 10^4^ cells per well in serum-free RPMI-1640 for cell survival assay or 5 × 10^3^ per well in RPMI-1640 supplemented with 1% FBS for cell growth assay) were seeded in 96-well plates and incubated at 37 °C. They were then treated with 100 ng/mL recombinant human insulin-like growth factor binding protein 2 (IGFBP2) (rhIGFBP2, ab63223; Abcam). The siRNA-transfected cells were not treated with rhIGFBP2. After 24, 48, and 72 h, CellTiter 96 Aqueous One Solution Reagent (Promega, Madison, WI, USA) was added to the cells, and the absorbance at 492 nm was measured using a microplate reader (Infinite 200 PRO).

### 2.9. Transwell Migration and Invasion Assay

For the migration assay, 1 × 10^5^ tumor cells were seeded in an 8.0 μm pore size insert (BD Falcon) containing RPMI-1640 with 0.1% FBS. For the invasion assay, 3 × 10^5^ tumor cells were seeded in the inserts of a Corning BioCoat^TM^ Matrigel Invasion Chamber (Corning, Tewksbury, MA, USA) containing RPMI-1640 with 0.1% FBS. The upper inserts, in which TE-8, TE-9, and TE-15 were seeded, were exposed to the lower chambers to which we added RPMI-1640 containing 0.1% FBS with/without rhIGFBP2. The inhibitors of PI3K (LY294002; Cell Signaling Technology), MEK1/2 (PD98059; Cell Signaling Technology), and NFκB (Bay117082; Sigma-Aldrich) were added in the upper inserts. The upper inserts with TE-10 and TE-11 transfected with siRNA were exposed to the lower chambers containing RPMI-1640 with 1.0% FBS. As for the Transwell assay to investigate the effect of the co-culture, 5 × 10^4^ MSCs or the three types of CAF-like cells in RPMI with 0.1% FBS were seeded in the lower chamber with/without Human IGFBP2 Antibody (2.5 μg/mL, AF674; R&D systems) or Normal Goat IgG Control (2.5 μg/mL, AB-108-C; R&D systems) as the negative control. The cells were incubated for 48 h at 37 °C in a CO_2_ incubator. Then, the cells remaining on the upper surface of the membrane were removed with a cotton swab. The cells that had migrated onto the lower surface of the membrane were stained using the Diff-Quik staining kit (Sysmex, Kobe, Japan) and counted.

### 2.10. Tissue Samples

Sixty-nine surgically resected human ESCC tissues collected between 2005 and 2010 at the Kobe University Hospital (Kobe, Japan) were used. None of the patients received any neoadjuvant chemotherapy or radiotherapy before the surgery. All study participants provided informed consent. The Institutional Review Board of Kobe University (B210103) approved all study protocols, and the study was conducted following the guidelines of the 1964 Declaration of Helsinki. The surgically resected samples were fixed using 10% formalin and embedded in paraffin. All samples were categorized using the Japanese Classification of Esophageal Cancer proposed by the Japan Esophageal Society and the TNM classification of Malignant Tumours proposed by the Union for International Cancer Control [21,22].

### 2.11. Immunofluorescence

TE-10 and TE-11 cells transfected with siNC (TE-10 siNC and TE-11 siNC) or siMT2A (TE-10 siMT2A and TE-11 siMT2A) were seeded on coverslips. The cells were then fixed with 100% methanol at −20 °C and incubated at 4 °C overnight with the primary antibodies: mouse antibody against E-cadherin (#610181; BD Biosciences, San Jose, CA, USA) and rabbit antibody against β-catenin (#8480; Cell Signaling Technology). After incubation and washing, the cells were incubated with Alexa Fluor 488-conjugated donkey anti-rabbit secondary antibody (Jackson ImmunoResearch Laboratories, West Grove, PA, USA) and Cy3-conjugated donkey anti-mouse secondary antibody (Jackson ImmunoResearch Laboratories) at room temperature for 1 h. DAPI (#340-07971; Wako) was used to stain the nuclei. Images were obtained using a Zeiss LSM 700 laser-scanning microscope and analyzed using the LSM ZEN 2009 software (Carl Zeiss, Oberkochen, Germany).

### 2.12. Immunohistochemistry

Immunohistochemistry was performed using the Leica BOND-MAX automated system and the BOND Polymer Refine Detection Kit (Leica Biosystems, Bannockburn, IL, USA). ESCC tissue sections of 4 μm thickness were incubated at room temperature in the Bond™ Epitope Retrieval Solution 2 (#AR9640; Leica Biosystems) for 10 min. Rabbit MT2A antibody (SAB4300966; Sigma-Aldrich) was used as the primary antibody. We used human liver tissue, which generally highly expresses MT2A, for the positive control and normal rabbit IgG (sc-20271; Santa Cruz Biotechnology) for the negative control (Figure S2). We evaluated the MT2A staining intensity of spindle cells (representative of CAFs) in the cancer stroma, as well as cells in the cancer nest (ESCC cells). MT2A expression levels were scored on a scale from 0 to 3, based on intensity. In the cancer stroma, if no positive cells were observed, the score attributed was 0. Further, the intensity of positive cells was compared with that of vascular endothelial cells. A relatively weaker signal was scored as 1, an equivalent signal as 2, and a stronger signal as 3. For ESCC cells in cancer nest, a score of 0 denotes a weaker signal, 1 implies an equivalent signal, and 2 represents a stronger signal intensity compared to that of normal epithelial cells. A score of 3 was assigned to intensities higher than 2. The scores were classified as high (score 3 in the cancer stroma and scores 2–3 in the cancer nest) and low (scores 0–2 in the cancer stroma and scores 0–1 in the cancer nest). The immunostaining evaluations were performed by two pathologists (Y.-i.K. and H.Y.) and one surgeon (M.S. (Masaki Shimizu)).

### 2.13. Statistical Analysis

The in vitro experiments were conducted in triplicate and independently performed three times. The results of the in vitro experiments are expressed as the mean ± standard error of mean (SEM), and the statistical significance was analyzed using the two-tailed Student’s *t*-test. The correlations between the clinicopathological factors and immunohistochemistry results were evaluated using *χ*^2^-tests. Overall survival (OS), disease-free survival (DFS), and cancer-specific survival (CSS) were visualized with Kaplan–Meier curves and analyzed using the log-rank test. A *p*-value < 0.05 was considered significant. Statistical analyses were carried out using SPSS Statistics ver. 22 (IBM, Chicago, IL, USA).

## 3. Results

### 3.1. High Expression of MT2A in CAF-Like Cells

**Figure 1:**
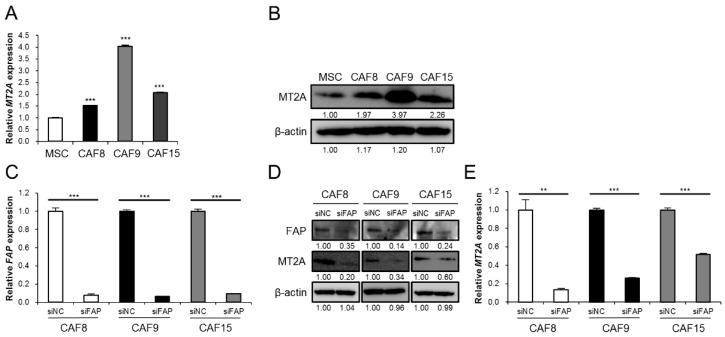
FAP regulates MT2A expression upstream in CAF-like cells. (**A**,**B**). The expression of *MT2A* mRNA and MT2A protein in mesenchymal stem cells (MSCs), CAF8, CAF9, and CAF15 cells was detected by quantitative real-time-PCR (qRT-PCR) (**A**) and Western blotting (**B**). After Western blotting, the normalized relative expression fold-change were calculated using the ImageJ software, and the values were arbitrarily set as 1.00 for control MSCs. (**C**–**E**). qRT-PCR (**C,E**) and Western blotting (**D**) to confirm FAP knockdown and change in the expression of MT2A. The three types of CAF-like cells were transfected with siRNA targeting FAP (siFAP) and the negative control siRNA (siNC). After Western blotting, the normalized relative expression fold-change were calculated using the ImageJ software, and the values were arbitrarily set as 1.00 for cells transfected with control siNC. Data are presented as mean ± SEM (** *p* < 0.01, *** *p* < 0.001).

To explore the role of CAFs in the ESCC tumor microenvironment, we established MSC-derived CAF-like cells by indirect co-culture of MSCs with ESCC cells. In a previous study, we compared MSCs and CAF-like cells by cDNA microarray analysis and found that, in the CAF-like cells, *MT2A* was the most upregulated among the differentially expressed genes [17]. Here, qRT-PCR and Western blotting confirmed that the *MT2A* mRNA and MT2A protein were highly expressed in all the three types of CAF-like cells (CAF8, CAF9, and CAF15) (Figure 1A,B and Figure S8A). To further investigate whether the CAF marker FAP regulates MT2A expression upstream, we knocked down *FAP* or *MT2A* in the CAF-like cells using siRNA. Knockdown of *FAP* in CAF8, CAF9, and CAF15 cells reduced the levels of both *MT2A* mRNA and MT2A protein (Figure 1C–E and Figure S8B), but knockdown of *MT2A* in the CAF-like cells did not reduce the expression of FAP (Figure S3A,B and Figure S8J).

### 3.2. MT2A Induces Expression and Secretion of IGFBP2 in CAF-Like Cells

**Figure 2:**
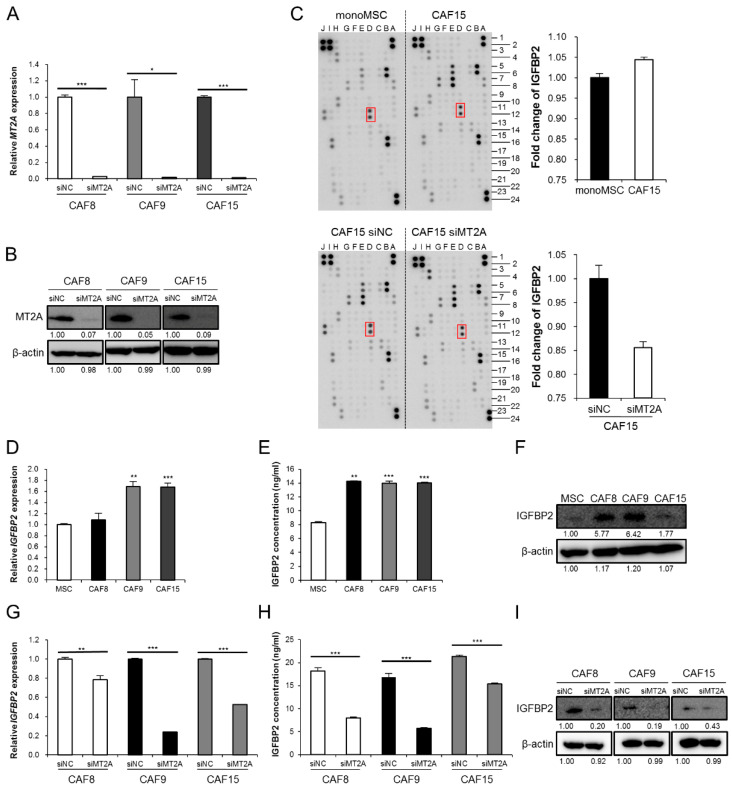
MT2A promotes the expression and secretion of IGFBP2 in CAF-like cells. (**A**,**B**) Three types of CAF-like cells (CAF8. CAF9, and CAF15) were transfected with siRNA targeting MT2A (siMT2A) and control siRNA (siNC). The knockdown was confirmed by qRT-PCR (**A**) and Western blotting (**B**). After Western blotting, the normalized relative expression fold-change was calculated using the ImageJ software, and the values were arbitrarily set as 1.00 for cells transfected with siNC. (**C**) Antibody array with monocultured MSC (monoMSC), CAF15, siNC-transfected CAF15 and siMT2A-transfected CAF15 conditioned medium. The intensity of insulin-like growth factor binding protein 2 (IGFBP2) was quantified using the public domain software ImageJ, normalized to a positive control reference spot. (**D**–**F**) Expression levels and secretory concentrations of IGFBP2 in CAF8, CAF9, and CAF15 cells were compared with those in MSCs using qRT-PCR (**D**), ELISA (**E**), and Western blotting (**F**). After Western blotting, the normalized relative expression fold-change was calculated using the ImageJ software, and the values were arbitrarily set as 1.00 for control MSCs. (**G**) Expression of *IGFBP2* was detected by qRT-PCR in CAF8, CAF9, and CAF15 cells transfected with siNC and siMT2A. (**H**) The secretory concentrations of IGFBP2 were detected by ELISA in CAF8, CAF9, and CAF15 cells transfected with siNC and siMT2A. (**I**) Western blotting for IGFBP2 in CAF8, CAF9, and CAF15 cells transfected with siNC and siMT2A. After Western blotting, the normalized relative expression fold-change was calculated using the ImageJ software, and the values were arbitrarily set as 1.00 for cells transfected with siNC. Data are presented as mean ± SEM (* *p* < 0.05, ** *p* < 0.01, *** *p* < 0.001).

To investigate the function of MT2A in CAF-like cells, we silenced *MT2A* by siRNA and confirmed a decrease in the levels of MT2A in the three types of CAF-like cells (Figure 2A,B and Figure S8C). Some reports suggest that MT2A is associated with the activation of intracellular signaling molecules such as NFκB, one of the regulators of cytokine expression. Therefore, we hypothesized that MT2A in CAFs regulates the secretion of tumor-promoting humoral factors by activating specific intracellular signaling pathways. To identify humoral factors regulated by MT2A in CAF-like cells, we performed an antibody array with CM of monoMSC, CAF15, CAF15 siNC, and CAF15 siMT2A. We observed an increase in IGFBP2 levels in CAF15 compared with those in monoMSCs and a decrease in CAF15 siMT2A compared with those in CAF15 siNC (Figure 2C and Figure S4). We then confirmed that the three types of CAF-like cells expressed and secreted higher levels of *IGFBP2* mRNA and IGFBP2 protein than MSCs, using qRT-PCR, ELISA, and Western blotting (Figure 2D–F and Figure S8D). Furthermore, the knockdown of *MT2A* in the CAF-like cells reduced the expression and secretion of IGFBP2, using qRT-PCR, ELISA, and Western blotting (Figure 2G–I and Figure S8E).

### 3.3. IGFBP2 Secreted by CAF-Like Cells Promotes Migration and Invasion of ESCC Cell Lines

**Figure 3:**
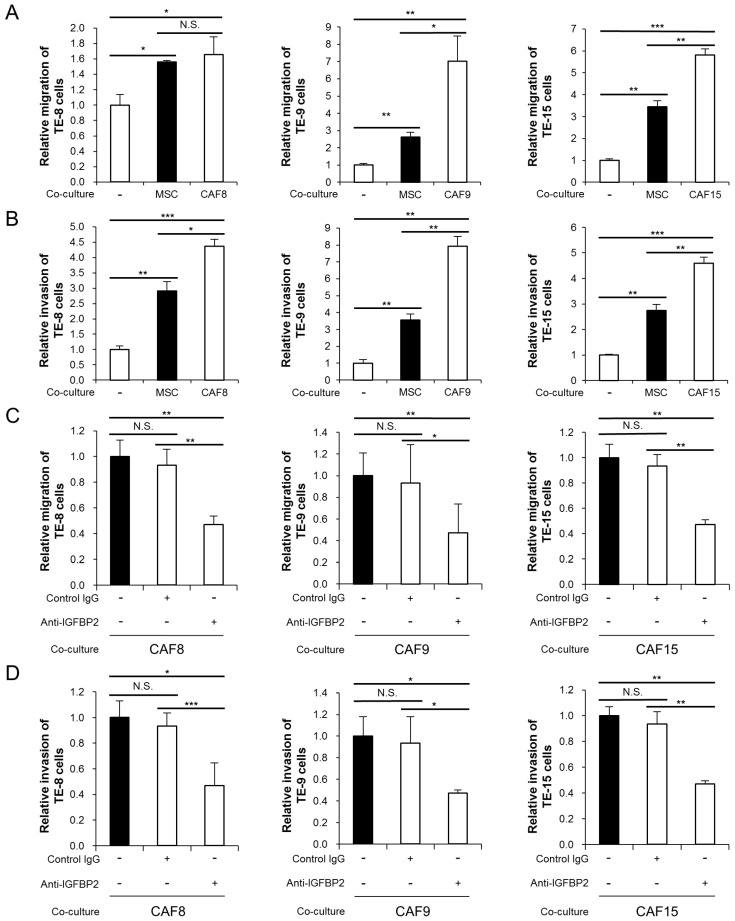
Anti-IGFBP2 neutralizing antibody suppressed the CAF-like cell-induced migration and invasion in three esophageal squamous cell carcinoma (ESCC) cell lines. (**A**,**B**) Transwell migration (**A**) and invasion (**B**) assays of TE-8, TE-9, and TE-15 ESCC cell lines co-cultured with MSCs or CAF-like cells compared with those of monocultured ESCC cells. Migrating and invading cells on the bottom of the upper chamber were counted in five randomly selected fields. (**C**,**D**) Transwell migration (**C**) and invasion (**D**) assays of ESCC cell lines co-cultured with CAF-like cells and with the anti-IGFBP2 neutralizing antibody or control goat IgG antibody, compared with those without antibody. Migrating and invading cells on the bottom of the upper chamber were counted in five randomly selected fields. Data are presented as mean ± SEM (* *p* < 0.05, ** *p* < 0.01, *** *p* < 0.001, N.S. not significant).

In our previous study, the three types of CAF-like cells (CAF8, CAF9, and CAF15) enhanced the migration and invasiveness of three types of ESCC cell lines (TE-8, TE-9, and TE-15, respectively) [17]. In this study, we found that while MSCs also enhance the migration and invasiveness of the ESCC cell lines, the effects of CAF-like cells exceed those of MSCs (Figure 3A,B). We hypothesized that IGFBP2 secreted from the CAF-like cells was responsible for these enhancement effects. Subsequently, the addition of a neutralizing antibody against IGFBP2 during the co-culture significantly suppressed the CAF-like cell-induced migration and invasion in the three ESCC cell lines (Figure 3C,D).

### 3.4. IGFBP2 Promotes Migration and Invasion through Akt, Erk, and NFκB Signaling Pathways in ESCC Cell Lines

**Figure 4:**
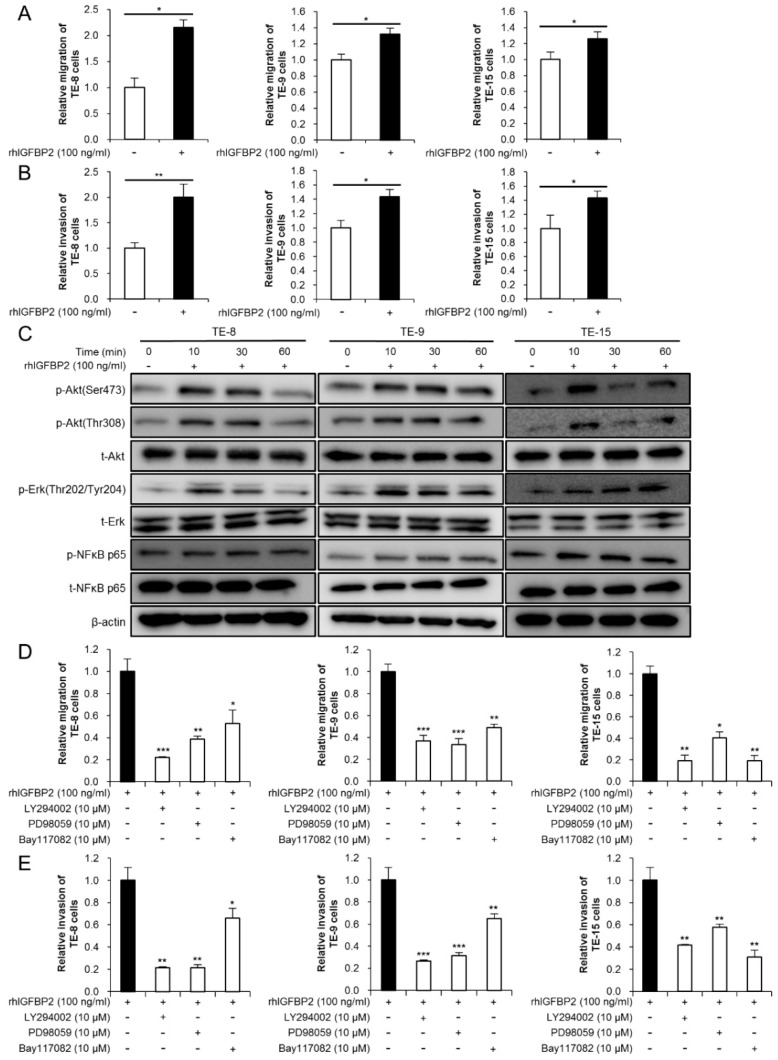
IGFBP2 promoted migration and invasion in ESCC cell lines through Akt, Erk, and NFκB signaling pathways. (**A**) Transwell migration assay of three ESCC cell lines stimulated with recombinant human IGFBP2 (rhIGFBP2). RhIGFBP2 was added to the lower chamber at a concentration of 100 ng/mL. Migrating cells on the bottom of the upper chamber were counted in five randomly selected fields. (**B**) Transwell invasion assay of three ESCC cell lines stimulated with rhIGFBP2. RhIGFBP2 was added to the lower chamber at a concentration of 100 ng/mL. Invading cells on the bottom of the upper chamber were counted in five randomly selected fields. (**C**) Western blotting to confirm the changes in the levels of total and phosphorylated Akt, Erk, and NFκB for 0, 10, 30, and 60 min after stimulation with rhIGFBP2 (100 ng/mL). TE-8, TE-9, and TE-15 cells were seeded on the day before protein extraction and incubated overnight in serum-free RPMI-1640. (**D**,**E**) Transwell migration (**D**) and invasion (**E**) assay of ESCC cell lines with inhibitors of phosphatidylinositol 3-kinase (PI3K inhibitor; LY294002, 10 μM), MEK1/2 (PD98059, 10 μM), and NFκB (Bay117082, 10 μM), stimulated with rhIGFBP2. Data are presented as mean ± SEM (* *p* < 0.05, ** *p* < 0.01, *** *p* < 0.001).

We stimulated TE-8, TE-9, and TE-15 with rhIGFBP2 to investigate the effect of IGFBP2 on malignancy in ESCC cell lines. The MTS assay demonstrated that rhIGFBP2 did not affect the cell growth and survival of the ESCC cell lines (Figure S5). However, rhIGFBP2 significantly promoted the migration and invasiveness of TE-8, TE-9, and TE-15 (Figure 4A,B). To identify the intracellular signaling pathways involved in these changes, we performed Western blotting 10, 30, and 60 min after stimulating the three ESCC cell lines with rhIGFBP2. Western blotting revealed that rhIGFBP2 induced the phosphorylation of Akt, Erk, and NFκB 10–30 min after its addition (Figure 4C and Figure S9). The inhibitors of PI3K (LY294002), MEK1/2 (PD98059), and NFκB (Bay1170982) suppressed rhIGFBP2-induced migration and invasion by TE-8, TE-9, and TE-15 cells (Figure 4D,E).

### 3.5. High Expression of MT2A in ESCC Cells Promotes Malignant Phenotype

**Figure 5:**
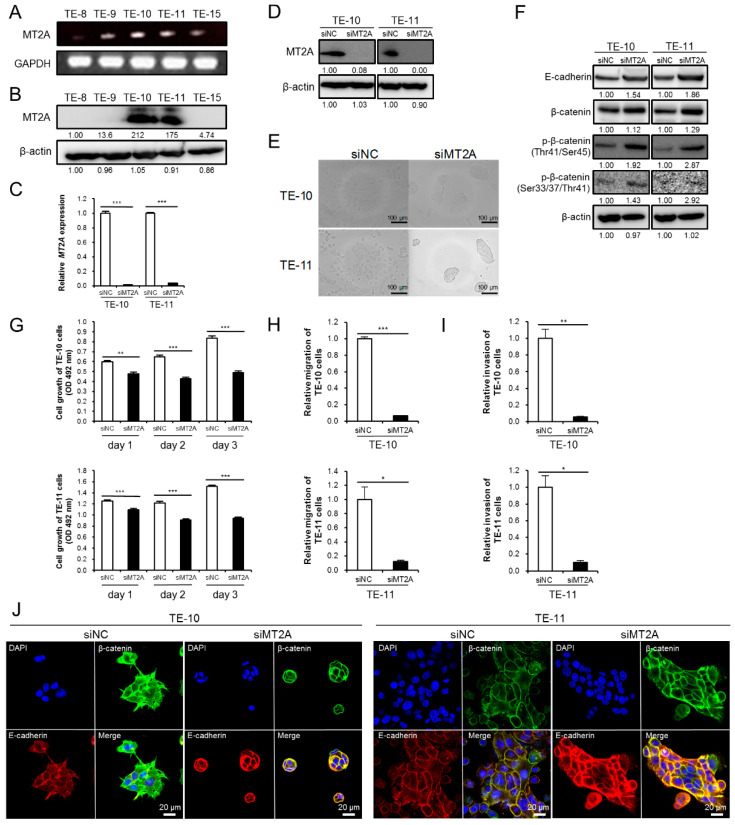
The high expression of MT2A in ESCC cells promoted their growth, migration, and invasiveness, possibly due to the downregulation of E-cadherin. (**A**) Expression of MT2A mRNA in five ESCC cell lines, TE-8, TE-9, TE-10, TE-11, and TE-15, evaluated by RT-PCR. *GAPDH* was used as the internal control. (**B**) Western blotting to confirm the expression of MT2A protein in the five ESCC cell lines. (**C**,**D**) TE-10 and TE-11 were transfected with siRNA against MT2A (siMT2A) and control siRNA (siNC). MT2A knockdown was confirmed by qRT-PCR (**C**) and Western blotting (**D**) in TE-10 and TE-11. After Western blotting, the normalized relative expression fold-change was calculated using the ImageJ software, and the values were arbitrarily set as 1.00 for the cells transfected with siNC. (**E**) Changes in the cell morphology of ESCC cell lines transfected with siNC or siMT2A under a phase contrast microscope. (**F**) Western blotting for expression levels of E-cadherin and phosphorylation levels of β-catenin in siMT2A-treated TE-10 and TE-11 cells, compared with those in cells treated with siNC. The normalized relative expression fold-change was calculated using the ImageJ software, and the values were arbitrarily set as 1.00 for cells transfected with siNC. (**G**) MTS assay for cell growth in TE-10 and TE-11 cells treated with siMT2A or siNC. Each ESCC cell line transfected with siNC and siMT2A was seeded in 96-well plates at 5 × 10^3^ cells per well with RPMI-1640 + 1% FBS. The cell growth was evaluated after 24, 48, and 72 h. (**H**,**I**) Transwell migration (**H**) and invasion (**I**) assay of ESCC cell lines transfected with siNC and siMT2A. (**J**) Double immunofluorescence was performed using anti-β-catenin (green) and anti-E-cadherin (red) antibodies in the ESCC cell lines transfected with siNC and siMT2A. Cell nuclei were stained with DAPI (blue). Data are presented as mean ± SEM (* *p* < 0.05, ** *p* < 0.01, *** *p* < 0.001).

We then explored the effect of MT2A in ESCC cell lines. We first assessed the degree of MT2A expression in five ESCC cell lines (TE-8, TE-9, TE-10, TE-11, and TE-15) using RT-PCR and Western blotting and observed that MT2A was highly expressed in TE-10 and TE-11 (Figure 5A,B and Figure S8F). To explore the functional role of MT2A in the ESCC cell lines, we silenced *MT2A* in TE-10 and TE-11 using siRNA (TE-10 and TE-11 siMT2A; Figure 5C,D and Figure S8G), and found remarkable changes in the cell morphology; the cells displayed a rounder shape and had proliferated more densely (Figure 5E). We speculated that these changes were related to cell adhesion and malignancy. E-cadherin is a transmembrane protein that strengthens cell adhesion when β-catenin binds to its cytoplasmic domain [23]. Western blotting showed an increased expression of E-cadherin and an increase in phosphorylated β-catenin (Figure 5F and Figure S8H). To further confirm whether MT2A knockdown in TE-10 and TE-11 cells can affect their malignant phenotype, we first performed an MTS assay for cell growth. The MTS assay demonstrated that cell growth was significantly suppressed in TE-10 and TE-11 after siMT2A transfection (Figure 5G). Next, we performed a Transwell assay for migration and invasion, and observed that siMT2A suppressed the migration and invasiveness of TE-10 and TE-11 cells (Figure 5H,I). Furthermore, double immunofluorescence using anti-E-cadherin and anti-β-catenin antibodies, to confirm the expression and localization of E-cadherin and β-catenin in the ESCC cell lines treated with siNC or siMT2A, revealed an enhancement in the co-localization of E-cadherin and β-catenin in both the ESCC cells transfected with siMT2A compared with that in cells transfected with siNC (Figure 5J). These results suggest that MT2A may promote a malignant phenotype in ESCC cells by decreasing the expression of E-cadherin.

### 3.6. High Expression Levels of MT2A in the Cancer Stroma and Cancer Nest Correlate with Poor Prognosis of ESCC Patients

**Figure 6:**
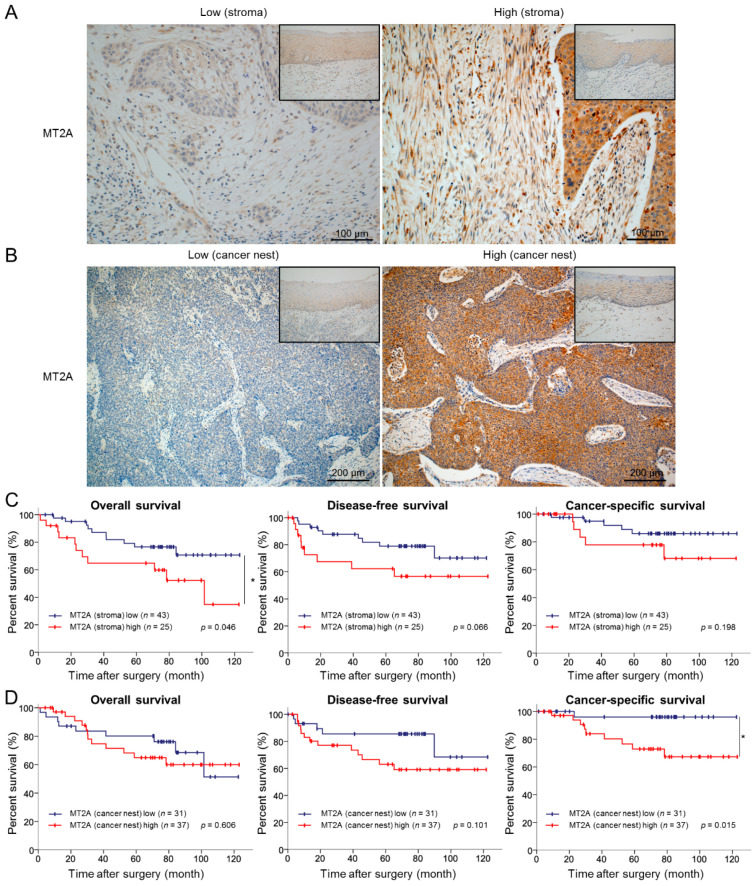
High expression levels of MT2A in the cancer stroma and cancer nest correlated to a poor prognosis of ESCC patients. (**A**) Immunohistochemical staining for MT2A in the cancer stroma of 69 human ESCC tissues. Representative immunostaining images show low intensity (left) and high intensity (right) with corresponding normal esophageal squamous epithelia (insets). Scale bars = 100 μm. (**B**) Immunohistochemical staining for MT2A in the cancer nest of 69 human ESCC tissues. Representative immunostaining images show low intensity (left) and high intensity (right) with corresponding normal esophageal squamous epithelia (insets). Scale bars = 200 μm. (**C**) Kaplan–Meier curves for overall survival, disease-free survival, and cancer-specific survival in 68 ESCC patients stratified into two groups based on MT2A expression levels in the cancer stroma: MT2A low cases (*n* = 43) and MT2A high cases (*n* = 25). (**D**) Kaplan–Meier curves for overall survival, disease-free survival, and cancer-specific survival in 68 ESCC patients stratified into two groups based on MT2A expression levels in the cancer nest: MT2A low cases (*n* = 31) and MT2A high cases (*n* = 37). Data are analyzed by log-rank test (* *p* < 0.05).

To investigate whether the high expression of MT2A in ESCC tissues correlates to the prognosis in ESCC patients, we evaluated the MT2A expression levels in ESCC tissues of 69 patients by immunohistochemistry. MT2A immunoreactivities of spindle cells in the cancer stroma (representative of CAFs) and cancer cells in the cancer nest (representative of ESCC cells) were examined, and the patients were divided into two groups: high expression and low expression of MT2A (Figure 6A,B). We then explored a relationship between MT2A expression and clinicopathological factors. High expression of MT2A in the cancer stroma was significantly associated with the depth of tumor invasion (*p* = 0.001), lymphatic vessel invasion (*p* < 0.001), blood vessel invasion (*p* = 0.008), lymph node metastasis (*p* = 0.031), pathological stage (*p* = 0.031), expression of alpha-smooth muscle actin (αSMA; *p* < 0.001) and FAP (*p* < 0.001), and infiltrating numbers of CD163^+^ (*p* = 0.004) and CD204^+^ (*p* < 0.001) cells. In contrast, high expression of MT2A in the cancer nest was not associated with any of the clinicopathological factors; however, it tended to correlate with the depth of tumor invasion (*p* = 0.07) (Table 1). We were then able to follow up with 68 patients to evaluate their disease outcome. The Kaplan–Meier analysis indicated that patients with a high expression of MT2A in the cancer stroma had significantly shorter OS (*p* = 0.046) and tended to have a shorter DFS (*p* = 0.066), while patients with a high expression of MT2A in the cancer nest had a significantly shorter CSS (*p* = 0.015) (Figure 6C,D).

## 4. Discussion

Here, we demonstrated that MT2A, one of the metallothionein (MT) isoforms, was highly expressed in three types of CAF-like cells that were induced by co-culture of MSCs with three ESCC cell lines, compared with that in monocultured MSCs. MTs are low molecular weight proteins (ranging from 6000 to 7000 Da) rich in cysteine-derived thiol groups. There are four major MT isoforms: MT1, MT2, MT3, and MT4. MT1 and MT2 are present in almost all mammalian organs, especially in the liver and kidneys [25]. *MT2*, which encodes a single protein MT2A, is the most expressed MT gene in humans [25]. Because MT can bind to heavy metal ions such as zinc in molecules, it is considered to be involved in maintaining the homeostasis of essential trace elements and detoxifying heavy metal ions. It was also reported that MTs could eliminate toxic radical species by their reaction with the thiol groups unbinding to heavy metal ions [25,26]. The reported roles of MTs in cancer cells have been contradictory. While Zhao et al. demonstrated that the direct interaction of MT2A with the BRCA1-associated RING domain 1 (BARD1) and MT2A-BARD1/BRCA1 axis promoted oxaliplatin resistance in colorectal cancer cells [27], another study reported that *MT2A* knockdown led to a high rate of apoptosis induced by cisplatin treatment in malignant pleural mesothelioma [28]. Furthermore, Tekur et al. demonstrated that ribozyme-induced downregulation of MT2A promoted apoptosis in human prostate and ovarian cancer cells [29]. These reports suggest that MT2A plays a role in chemoresistance and evasion of apoptosis as a tumor promoter. In contrast, MT2A was also shown to suppress tumor progression by inhibiting NFκB in gastric cancer [30]. It was also reported that the MT1M isoform has a tumor suppressive function in ESCC cell lines in vitro [31]. However, to the best of our knowledge, this is the first report on the role of MT2A in the ESCC microenvironment, including cancer cells and CAFs.

In this study, we confirmed that MT2A in CAFs modulates the expression and secretion of IGFBP2. It is known that MT2A also regulates the activity of transcription factors such as NFκB and AP-1 [32,33] and was reported to promote colorectal carcinogenesis through the interaction of phosphorylated Fas-associated death domain (FADD) and NFκB [34,35]. Yang et al. reported that zinc is an essential component in the DNA-binding function of NFκB, and MT2A might regulate the transcriptional activity of NFκB by supplying zinc [36]. It was also suggested that MT2A expression is associated with the expression of humoral factors such as VEGF [37]. Thus, a potential mechanism by which MT2A induces IGFBP2 could be that it serves as a zinc donor and regulates the activity of specific zinc-dependent transcription factors targeting IGFBP2.

IGFBP2 was initially identified as a secretory protein that binds to insulin growth factor 1 (IGF-1) and IGF-2 to regulate their activities [38,39]. Apart from binding to IGFs, IGFBP2 is also known to be involved in intracellular signal transduction [40]. In malignant tumors such as prostate cancer, high-grade glioma, and hepatocellular carcinoma, high expression of IGFBP2 was correlated to poor prognosis [41,42,43,44,45]. In this study, we confirmed that rhIGFBP2 promotes the migration and invasiveness of ESCC cell lines via the Akt, Erk, and NFκB signaling pathways. IGFBP2 contains an RGD motif through which it binds to integrin and affects cell proliferation and survival in high-grade glioma and hepatocellular carcinoma [43,46,47]. Additionally, it is known that IGFBP2, containing the nuclear localization signal sequence (NLS), binds to the epidermal growth factor receptor (EGFR) and facilitates its nuclear accumulation [44,48]. IGFBP2 also acts as a tumor promoter in glioma and pancreatic cancer by activating nuclear EGFR-STAT3 signaling [42,44,48]. Hence, IGFBP2 may activate intracellular signaling through receptors such as EGFR and integrin. Here, we could not identify the precise receptor involved in driving the effects of IGFBP2 in ESCC cells, which is a limitation of this study. Moreover, because the addition of a neutralizing antibody against IGFBP2 only partially suppressed the CAF-like cell-induced migration and invasiveness in three ESCC cell lines, we considered that the paracrine effects of CAF-like cells were not limited to the effects of IGFBP2. Our previous reports demonstrated that IL-6, CCL2, and PAI-1 derived from CAF-like cells also have tumor-promoting roles, including the induction of migration and invasiveness of ESCC cell lines [16,17].

In this study, we also demonstrated that high expression of MT2A in the cancer nest is associated with poor prognosis of ESCC patients. Therefore, we hypothesized that high expression of MT2A promotes malignant phenotypes in ESCC cell lines in vitro. We found that the MT2A expression varied among the five ESCC cell lines. It is reported that MT2A promotes the migration and invasiveness of cancer cells in breast cancer and mucoepidermoid cancer by regulating MMP9 expression [33,49]. To identify the function of MT2A in ESCC cells, we silenced *MT2A* using siRNA in two ESCC cell lines that highly express MT2A, TE-10 and TE-11. It was reported that MT2A reduces the activity of NFκB and suppresses the malignant phenotypes in gastric cancer [30]. Kaplan–Meier survival assays indicated that patients with a high expression level of MT2A in gastric cancer had a significantly better overall survival [30]. In our study, Western blotting showed that phosphorylated NFκB increased in TE-10 and TE-11 cells treated with siMT2A (Figure S6 and Figure S8K). This result suggests that MT2A in TE-10 and TE-11 inhibits the activity of NFκB, similar to that in gastric cancer. However, the knockdown of MT2A inhibited cell growth, migration, and invasiveness of TE-10 and TE-11 in our study. Additionally, ESCC patients with high MT2A expression in the cancer nest had a poor prognosis. These results are contrary to tumor suppressor roles of MT2A in gastric cancer. Hence, we considered that another pathway other than NFκB might be important in ESCC cells and focused on cell morphology changes in TE-10 and TE-11 cells transfected with siMT2A. We observed that these ESCC cells became round in shape and proliferated more densely. We then speculated that this change in cell morphology was due to an enhancement in cell adhesion and found increases in both the mRNA (Figure S7) and protein levels of E-cadherin, a cell adhesion molecule, in TE-10 and TE-11 cells treated with siMT2A, compared with those in cells treated with siNC. As the expression of E-cadherin is repressed by the zinc finger E-box binding homeobox (ZEB) protein belonging to the ZFH family, it is possible that MT2A regulates the expression of E-cadherin through a zinc finger protein, acting as a zinc donor [50]. The knockdown of *MT2A* inhibited growth, migration, and invasiveness of the ESCC cell lines. The binding of β-catenin to the cytoplasmic domain of the transmembrane E-cadherin strengthens cell–cell adhesion [51]. Further, β-catenin also functions as a transcription factor and requires Wnt signaling for its stabilization [23,51]. In the absence of stimulation by a Wnt signal, β-catenin is phosphorylated by CK1 (at Ser45) and GSK3β (at Ser33/Ser37/Thr41) into a transcriptionally inactive form [52]. Onder et al. demonstrated that E-cadherin loss-induced β-catenin translocation from the cytoplasm to the nucleus decreased the inactivating phosphorylation of β-catenin and promoted the migration, invasiveness, and survival of breast cancer cells [53]. Yang et al. reported that FOXP3 facilitated Wnt/β-catenin signaling by decreasing the expression of E-cadherin and promoted proliferation and metastasis in human non-small cell lung cancer [54]. Our study revealed that the knockdown of *MT2A* in ESCC cell lines increased the inactivating phosphorylation of β-catenin. Further, double fluorescent immunostaining displayed the cytoplasmic localization of β-catenin in the control cells and co-localization with E-cadherin on the cell membrane in siMT2A-transfected ESCC cell lines. From these results, we concluded that the high expression of E-cadherin, due to a loss of function in MT2A, led to the capture of β-catenin on the cell membrane and increased the levels of its transcriptionally inactive form, thereby inhibiting cell growth, migration, and invasiveness of ESCC cell lines. In other words, the high expression of MT2A in ESCC cells may promote tumor progression and malignancy through the E-cadherin/β-catenin signaling pathway.

In this study, the high expression of MT2A in the cancer nest tended to associate with the depth of tumor invasion (*p* = 0.07). This finding corresponds to the effect of MT2A on the migration and invasiveness of ESCC cell lines in vitro. Furthermore, the high expression of MT2A in the cancer stroma and cancer nest also correlated with poor prognosis of ESCC patients. To the best of our knowledge, our study is the first to report an association between MT2A immunoreactivity in the cancer stroma and poor prognosis of ESCC patients. It was also previously reported that the high expression of MT, including MT1 and MT2, in the cancer nest was associated with clinicopathological factors and poor prognosis based on immunohistochemical evaluation in ESCC tissues [55]. This result is similar to our findings. Therefore, MT2A expression may be used for the prognostic factor of ESCC.

A neutralizing antibody against IGFBP2 was reported to inhibit the malignant phenotype of glioma in vitro and in vivo [56,57]. Our investigation also showed that the addition of a neutralizing anti-IGFBP2 antibody suppressed migration and invasiveness of ESCC cells, induced by their co-culture with CAF-like cells. This indicates that the inhibition of IGFBP2 can be a novel therapeutic strategy for ESCC. Moreover, targeting *MT2A* using RNA interference could also be a potential therapeutic approach against ESCC, as it inhibited the development of a malignant phenotype in the ESCC cell lines. RNA interference as a treatment strategy is receiving widespread attention and is expected to be applied to cancer therapy [58,59]. However, as our findings were limited to in vitro experiments, further exploration of the functional roles of MT2A and IGFBP2 in vivo will be required.

## 5. Conclusions

In this study, we reported that high expression of MT2A in the cancer stroma and cancer nest is involved in tumor progression in the ESCC microenvironment. MT2A in CAFs promoted the expression and secretion of IGFBP2. The ESCC cells acquired a malignant phenotype through the paracrine activity of IGFBP2. Furthermore, MT2A in ESCC cells may regulate the E-cadherin/β-catenin signaling pathway to promote tumor progression. Our findings indicate that MT2A and IGFBP2 are potential novel therapeutic targets in ESCC.

## Figures and Tables

**Table 1:** Expression levels of MT2A in human ESCC tissues and their correlation with clinicopathological features.

Variable	Expression of MT2A in Cancer Stroma ^1^				Expression of MT2A in Cancer Nest ^1^			
	Number of Cases	Low	High		Number of Cases	Low	High	
		(*n* = 43)	(*n* = 26)	*p*-Value		(*n* = 31)	(*n* = 38)	*p*-Value
Age								
<65	32	20	12	0.997	32	13	19	0.504
≥65	37	23	14		37	18	19	
Sex								
Male	14	8	6	0.654	14	6	8	0.862
Female	55	35	20		55	25	30	
Histological grade ^2^								
HGIEN + WDSCC	15	11	4	0.320	15	8	7	0.459
MDSCC + PDSCC	54	32	22		54	23	31	
Depth of tumor invasion ^2^								
T1	48	36	12	0.001 **	48	25	23	0.071
T2+T3	21	7	14		21	6	15	
Lymphatic vessel invasion ^2^								
Negative	37	31	6	<0.001 ***	37	19	18	0.249
Positive	32	12	20		32	12	20	
Blood vessel invasion ^2^								
Negative	43	32	11	0.008 **	43	20	23	0.734
Positive	26	11	15		26	11	15	
Lymph node metastasis ^2^								
Negative	43	31	12	0.031 *	43	22	21	0.181
Positive	26	12	14		26	9	17	
Stage ^3^								
0 + I	38	28	10	0.031 *	38	19	19	0.348
II + III + IV	31	15	16		31	12	19	
Expression of αSMA ^4^								
Low	36	31	5	<0.001 ***	36	16	20	0.933
High	33	12	21		33	15	18	
Expression of FAP ^4^								
Low	39	33	6	<0.001 ***	39	19	20	0.470
High	30	10	20		30	12	18	
Expression of CD163 ^5^								
Low	34	27	7	0.004 **	34	16	18	0.726
High	35	16	19		35	15	20	
Expression of CD204 ^5^								
Low	34	31	3	<0.001 ***	34	16	18	0.726
High	35	12	23		35	15	20	

Data were analyzed by *χ^2^*-test; *p* < 0.05 was considered statistically significant: * *p* < 0.05; ** *p* < 0.01; *** *p* < 0.001. ^1^ The ESCC tissues were divided into two groups (high- and low-) based on the immunoreactivity intensity of MT2A in cancer stroma or cancer nest. ^2^ Based on the Japanese Classification of Esophageal Cancer 10th ed [21]. HGIEN, high-grade intraepithelial neoplasia; WDSCC, well-differentiated squamous cell carcinoma; MDSCC, moderately differentiated squamous cell carcinoma; PDSCC, poorly differentiated squamous cell carcinoma. T1, tumor invades mucosa and submucosa; T2, tumor invades muscularis propria; T3, tumor invades adventitia. ^3^ Based on the TNM classification 7th ed. by UICC [22]. ^4^ Immunoreactivity at the invasive front of ESCC was classified based on the staining area (high: >30%; low: ≤30%) [16]. ^5^ The median of CD163^+^ or CD204^+^ macrophage counts in cancer nests and stroma within the areas were used to classify the patients as low- and high-groups [24].

## Data Availability

The data presented in this study are available on request from the corresponding author.

## Supplementary Materials

The following are available online at https://www.mdpi.com/article/10.3390/cancers13184552/s1, Figure S1: CAF-like cells, MSCs co-cultured with ESCC cell lines, showed elevated FAP expression. (A,B) Quantitative real-time-PCR (qRT-PCR) (A) and Western blotting (B) showing the relative expression levels of *FAP* mRNA and FAP protein in MSCs and CAF-like cells. After Western blotting, the normalized relative expression fold-change were calculated using the ImageJ software, and the values were arbitrarily set as 1.00 for control MSCs. Data are presented as mean ± SEM (** *p* < 0.01).; Figure S2: Positive and negative controls for immunohistochemistry. (A) Immunohistochemical staining of normal liver tissue using rabbit MT2A antibody as the positive control (left) and normal rabbit IgG as the negative control (right). (B) Immunohistochemical staining of human esophageal squamous cell carcinoma tissue using normal rabbit IgG as the negative control. Scale bar = 100 μm.; Figure S3: Knockdown of *MT2A* in CAF-like cells did not reduce the expression of FAP. (A) CAF8, CAF9, and CAF15 cells were transfected with siRNA targeting *MT2A* (siMT2A) and the negative control siRNA (siNC). The expression levels of *FAP* mRNA in CAF8, CAF9, and CAF15 cells were compared between the cells transfected with siNC and those with siMT2A, by qRT-PCR. (B) Western blotting to investigate the effect of *MT2A* knockdown on FAP expression in the three types of CAF-like cells. The normalized relative expression fold-change was calculated using the Image J software, and the values were arbitrarily set as 1.00 for cells transfected with siNC. Data are presented as mean ± SEM (N.S. not significant).; Figure S4: The coordinate of capture antibodies in the Proteome Profiler Human XL Cytokine Array Kit.; Figure S5: IGFBP2 did not promote cell survival and growth in ESCC cell lines. (A,B) MTS assay for cell survival (A) and growth (B) in TE-8, TE-9, and TE-15 cells stimulated with recombinant human IGFBP2 (rhIGFBP2). Each ESCC cell line was seeded in 96-well plates at 1 × 10^4^ cells per well with serum-free RPMI for the cell survival assay and at 5 × 10^3^ cells per well with RPMI-1640 + 1% FBS for the cell growth assay. Evaluation of these assays was conducted after 48 h. Data are presented as mean ± SEM (N.S. not significant).; Figure S6: Western blotting to investigate the effect of *MT2A* knockdown on phosphorylation levels of NFκB in siMT2A-treated TE-10 and TE-11 cells, compared with those in cells treated with siNC. The normalized relative expression fold-change was calculated using the Image J software, and the values were arbitrarily set as 1.00 for cells transfected with siNC.; Figure S7: Knockdown of *MT2A* in ESCC cell lines increased the expression of *CDH1* mRNA. qRT-PCR to assess the expression levels of *CDH1* in TE-10 and TE-11 cells transfected with siMT2A and siNC. Data are presented as mean ± SEM (*** *p* < 0.001).; Figure S8: Uncropped the Western blotting images for Figure 1B,D, Figure 2B,F,I, Figure 5B,D,F, Figure S1B, Figure S3B, and Figure S6, corresponding to Figure S8A–K, respectively. The Western blotting membrane was cut and used for immunoblotting several times to identify other bands. Because prestained markers of three colors that do not contain chemiluminescent substances were used, the markers cannot be visualized in the raw data presented.; Figure S9: Uncropped the Western blotting images for Figure 4C. The Western blotting membranes were cut and used several times to identify other bands. Because prestained markers of three colors that do not contain chemiluminescent substances were used, the markers cannot be visualized in the raw data presented.
