# Supplementary material for: Metallothionein 2A Expression in Cancer-Associated Fibroblasts and Cancer Cells Promotes Esophageal Squamous Cell Carcinoma Progression
Source: Cancers (Basel). 2021 Sep 10;13(18):4552. doi: 10.3390/cancers13184552 (PMC8464741; doi:10.3390/cancers13184552)
Supplement: Supplementary file 1 [file cancers-13-04552-s001.zip › cancers-1345721-Suppl-New.pdf]

cancers MDPI

Supplementary Materials: Metallothionein 2A Expression in Cancer-Associated Fibroblasts and Cancer Cells Promotes Esophageal Squamous Cell Carcinoma Progression

Masaki Shimizu, Yu-ichiro Koma, Hiroki Sakamoto, Shuichi Tsukamoto, Yu Kitamura, Satoshi Urakami, Kohei Tanigawa, Takayuki Kodama, Nobuhide Higashino, Mari Nishio, Manabu Shigeoka, Yoshihiro Kakeji and Hiroshi Yokozaki

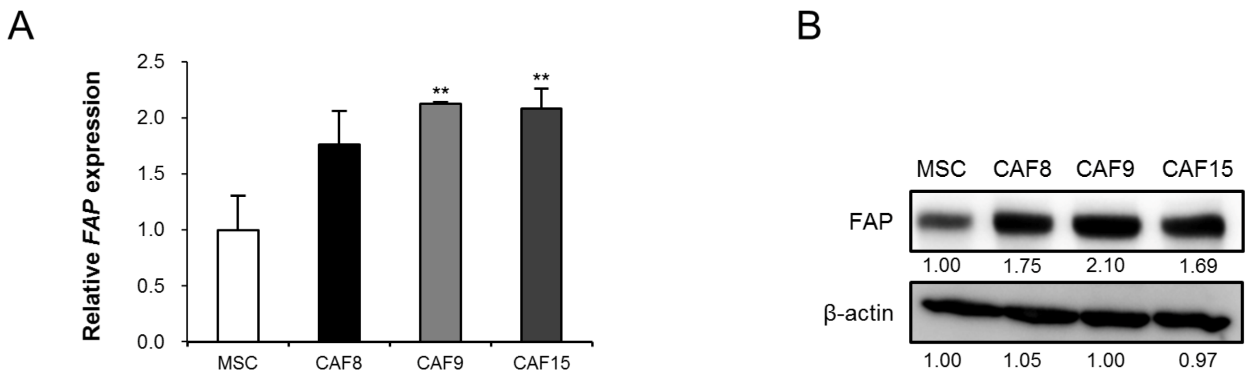

Figure S1. CAF-like cells, MSCs co-cultured with ESCC cell lines, showed elevated FAP expression. (**A, B**) Quantitative real-time-PCR (qRT-PCR) (A) and Western blotting (B) showing the relative expression levels of *FAP* mRNA and FAP protein in MSCs and CAF-like cells. After Western blotting, the normalized relative expression fold-change were calculated using the ImageJ software, and the values were arbitrarily set as 1.00 for control MSCs. Data are presented as mean ± SEM (** $p < 0.01$).

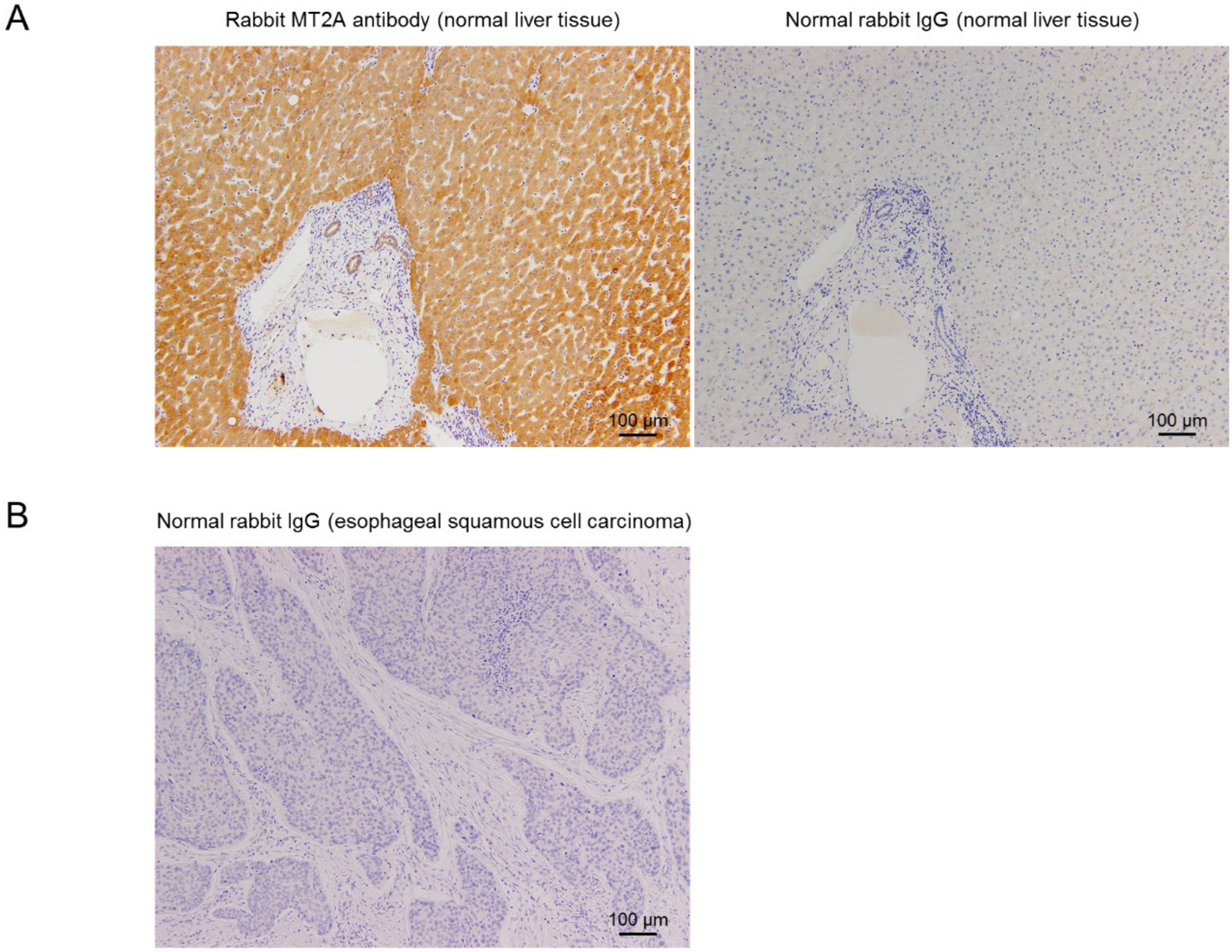

Figure S2. Positive and negative controls for immunohistochemistry. (**A**) Immunohistochemical staining of normal liver tissue using rabbit MT2A antibody as the positive control (left) and normal rabbit IgG as the negative control (right). (**B**) Immunohistochemical staining of human esophageal squamous cell carcinoma tissue using normal rabbit IgG as the negative control. Scale bar = 100 μm.

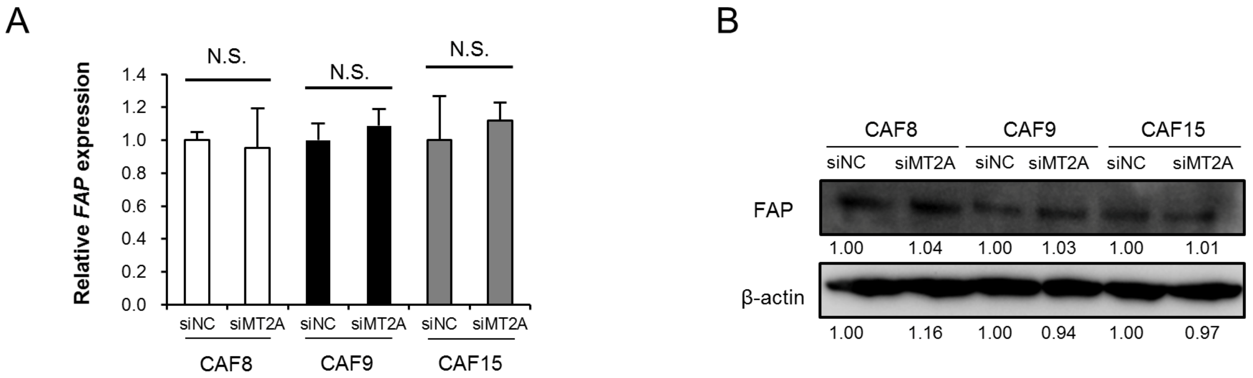

Figure S3. Knockdown of *MT2A* in CAF-like cells did not reduce the expression of FAP. (**A**) CAF8, CAF9, and CAF15 cells were transfected with siRNA targeting *MT2A* (siMT2A) and the negative control siRNA (siNC). The expression levels of *FAP* mRNA in CAF8, CAF9, and CAF15 cells were compared between the cells transfected with siNC and those with siMT2A, by qRT-PCR. (**B**) Western blotting to investigate the effect of *MT2A* knockdown on FAP expression in the three types of CAF-like cells. The normalized relative expression fold-change was calculated using the Image J software, and the values were arbitrarily set as 1.00 for cells transfected with siNC. Data are presented as mean ± SEM (N.S. not significant).

J	I	H	G	F	E	D	C	B	A	
Reference Spots	PAI-1	MPO	Leptin	IL-19	IL-4	CXCL1			Reference Spots	1<br>2
	ABP	OPN	LIF	IL-22	IL-5	GH	CXCL5	CD40L	Acrp30	3<br>4
VDB	IL1RL1	PDGF-AA	NGAL	IL-23	IL-6	HGF	ENG	YKL-40	ApoA1	5<br>6
CD31	CCL17	PDGF-AB/BB	CCL2	IL-24	IL-8	ICAM-1	CD178	CFD	Angiogenin	7<br>8
TIM-3	ITF	PTX3	CCL7	IL-27	IL-10	INF-γ	FGF-2	CRP	Ang-1	9<br>10
VCAM-1	CD71	CXCL4	M-CSF	IL-31	IL-11	IGFBP2	FGF-7	Cripto-1	Ang-2	11<br>12
	TGF-α	RAGE	MIF	IL-32	IL-12 p70	IGFBP3	FGF-19	CST3	BAFF	13<br>14
	TSP-1	CCL5	CXCL9	IL-33	IL-13	IL-1α	FLT3LG	Dkk-1	BDNF	15<br>16
	TNF-α	RBP-4	CCL3 /CCL4	IL-34	IL-15	IL-1β	G-CSF	CD26	C5/C5a	17<br>18
	uPAR	RLN2	CCL20	CXCL10	IL-16	IL-1ra	GDF-15	EGF	CD14	19<br>20
	VEGF	RETN	CCL19	CXCL11	IL-17A	IL-2	GM-CSF	CD147	CD30	21<br>22
Negative Control		CXCL12	MMP-9	PSA	IL-18 Bpa	IL-3			Reference Spots	23<br>24

Figure S4. The coordinate of capture antibodies in the Proteome Profiler Human XL Cytokine Array Kit.

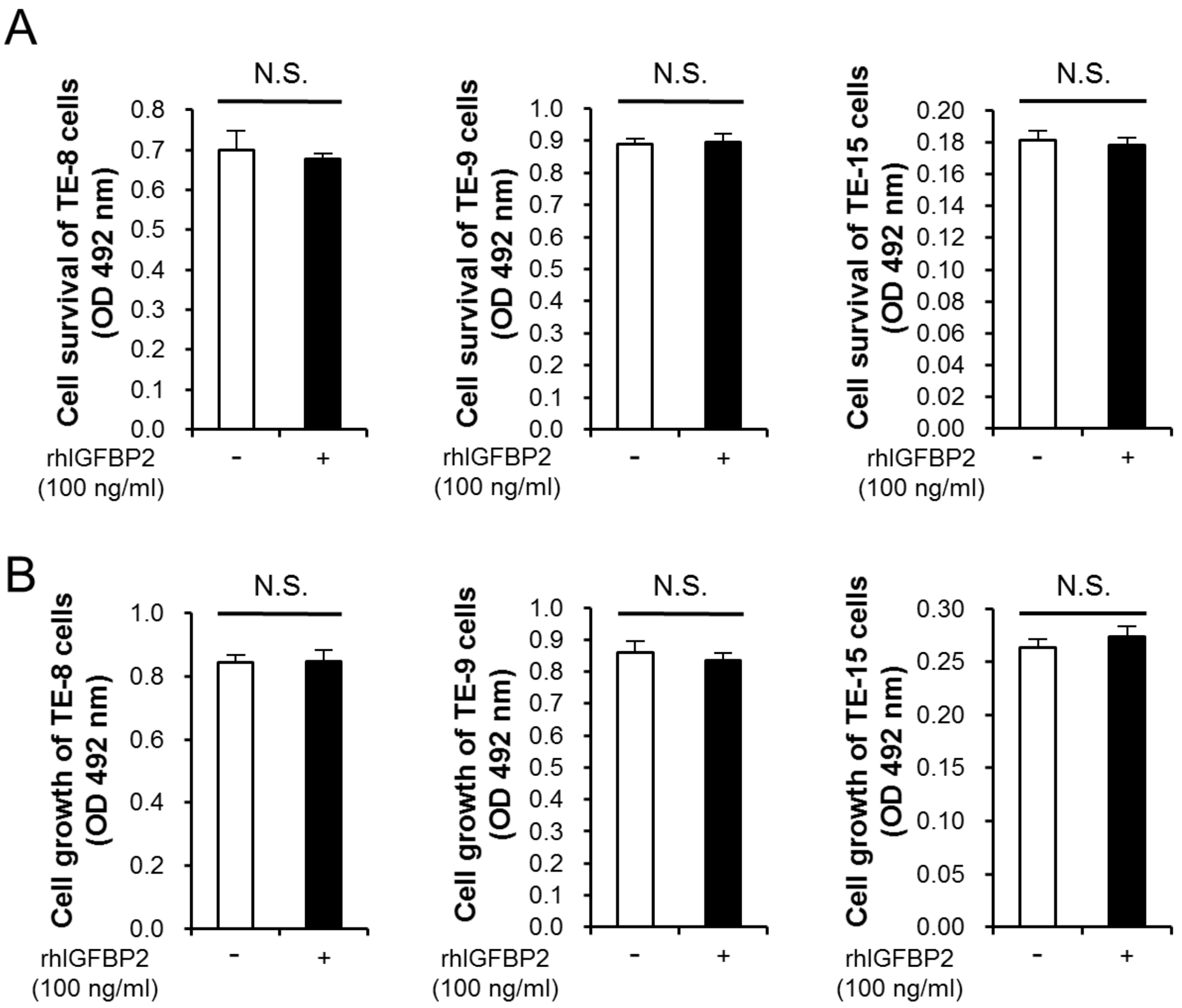

Figure S5. IGFBP2 did not promote cell survival and growth in ESCC cell lines. (**A**, **B**) MTS assay for cell survival (A) and growth (B) in TE-8, TE-9, and TE-15 cells stimulated with recombinant human IGFBP2 (rhIGFBP2). Each ESCC cell line was seeded in 96-well plates at 1×10^4 cells per well with serum-free RPMI for the cell survival assay and at 5×10^3 cells per well with RPMI-1640 + 1% FBS for the cell growth assay. Evaluation of these assays was conducted after 48 h. Data are presented as mean ± SEM (N.S. not significant).

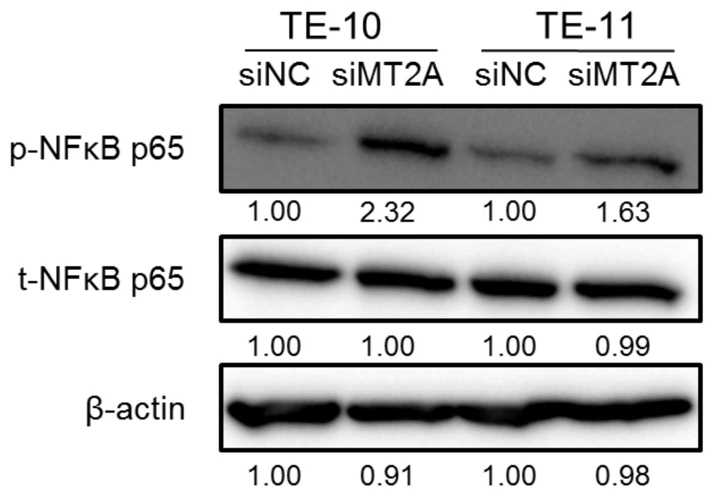

Figure S6. Western blotting to investigate the effect of *MT2A* knockdown on phosphorylation levels of NFκB in siMT2A-treated TE-10 and TE-11 cells, compared with those in cells treated with siNC. The normalized relative expression fold-change was calculated using the Image J software, and the values were arbitrarily set as 1.00 for cells transfected with siNC.

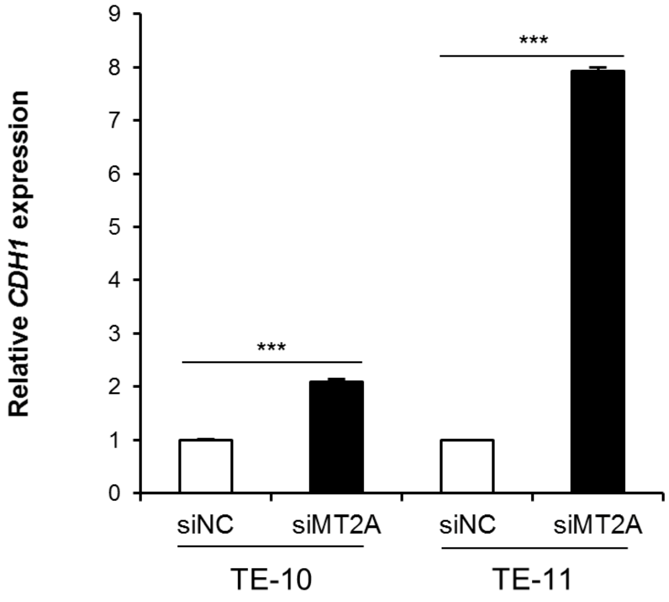

Figure S7. Knockdown of *MT2A* in ESCC cell lines increased the expression of *CDH1* mRNA. qRT-PCR to assess the expression levels of *CDH1* in TE-10 and TE-11 cells transfected with siMT2A and siNC. Data are presented as mean ± SEM (*** $p < 0.001$).

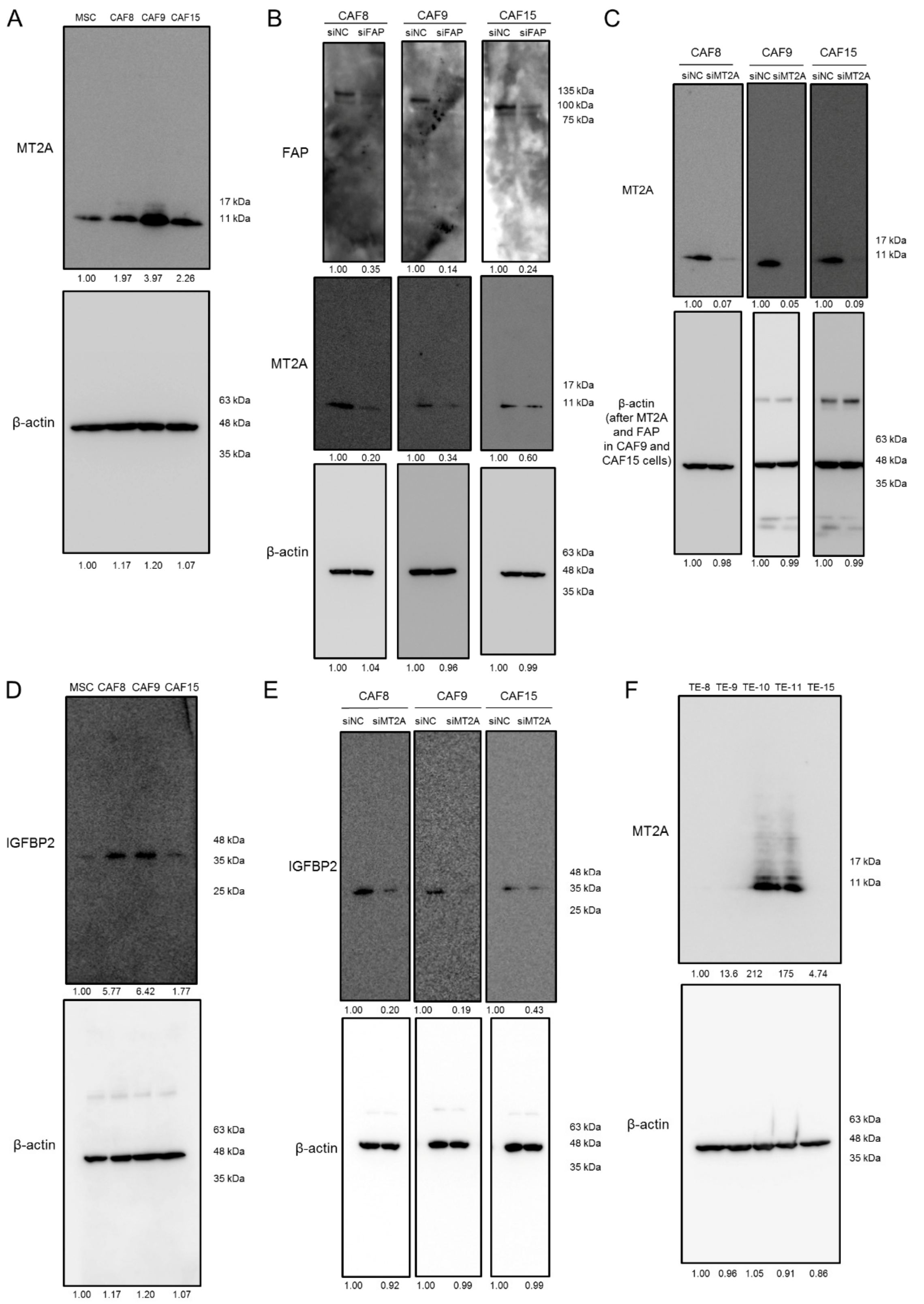
A
MSC CAF8 CAF9 CAF15
MT2A
17 kDa
11 kDa
1.00 1.97 3.97 2.26
β-actin
63 kDa
48 kDa
35 kDa
1.00 1.17 1.20 1.07
B
CAF8 CAF9 CAF15
siNC siFAP siNC siFAP siNC siFAP
FAP
135 kDa
100 kDa
75 kDa
1.00 0.35 1.00 0.14 1.00 0.24
MT2A
17 kDa
11 kDa
1.00 0.20 1.00 0.34 1.00 0.60
β-actin
63 kDa
48 kDa
35 kDa
1.00 1.04 1.00 0.96 1.00 0.99
C
CAF8 CAF9 CAF15
siNC siMT2A siNC siMT2A siNC siMT2A
MT2A
17 kDa
11 kDa
1.00 0.07 1.00 0.05 1.00 0.09
β-actin (after MT2A and FAP in CAF9 and CAF15 cells)
63 kDa
48 kDa
35 kDa
1.00 0.98 1.00 0.99 1.00 0.99
D
MSC CAF8 CAF9 CAF15
IGFBP2
48 kDa
35 kDa
25 kDa
1.00 5.77 6.42 1.77
β-actin
63 kDa
48 kDa
35 kDa
1.00 1.17 1.20 1.07
E
CAF8 CAF9 CAF15
siNC siMT2A siNC siMT2A siNC siMT2A
IGFBP2
48 kDa
35 kDa
25 kDa
1.00 0.20 1.00 0.19 1.00 0.43
β-actin
63 kDa
48 kDa
35 kDa
1.00 0.92 1.00 0.99 1.00 0.99
F
TE-8 TE-9 TE-10 TE-11 TE-15
MT2A
17 kDa
11 kDa
1.00 13.6 212 175 4.74
β-actin
63 kDa
48 kDa
35 kDa
1.00 0.96 1.05 0.91 0.86

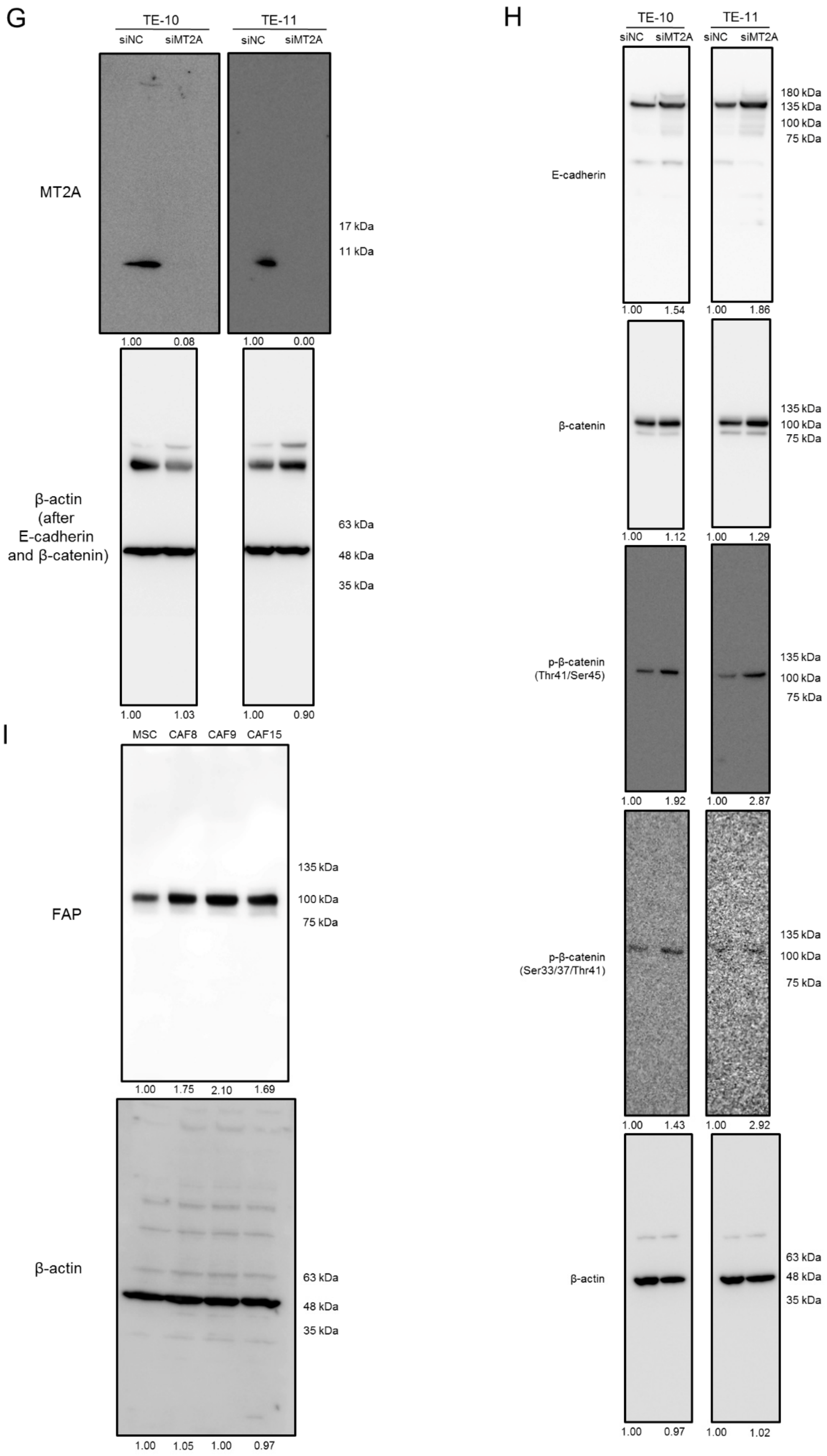
G
TE-10
TE-11
siNC
siMT2A
MT2A
17 kDa
11 kDa
1.00 0.08
1.00 0.00
β-actin (after E-cadherin and β-catenin)
63 kDa
48 kDa
35 kDa
1.00 1.03
1.00 0.90
H
E-cadherin
180 kDa
135 kDa
100 kDa
75 kDa
1.00 1.54
1.00 1.86
β-catenin
1.00 1.12
1.00 1.29
p-β-catenin (Thr41/Ser45)
1.00 1.92
1.00 2.87
p-β-catenin (Ser33/37/Thr41)
1.00 1.43
1.00 2.92
β-actin
1.00 0.97
1.00 1.02
I
MSC
CAF8
CAF9
CAF15
FAP
1.00 1.75 2.10 1.69
β-actin
1.00 1.05 1.00 0.97

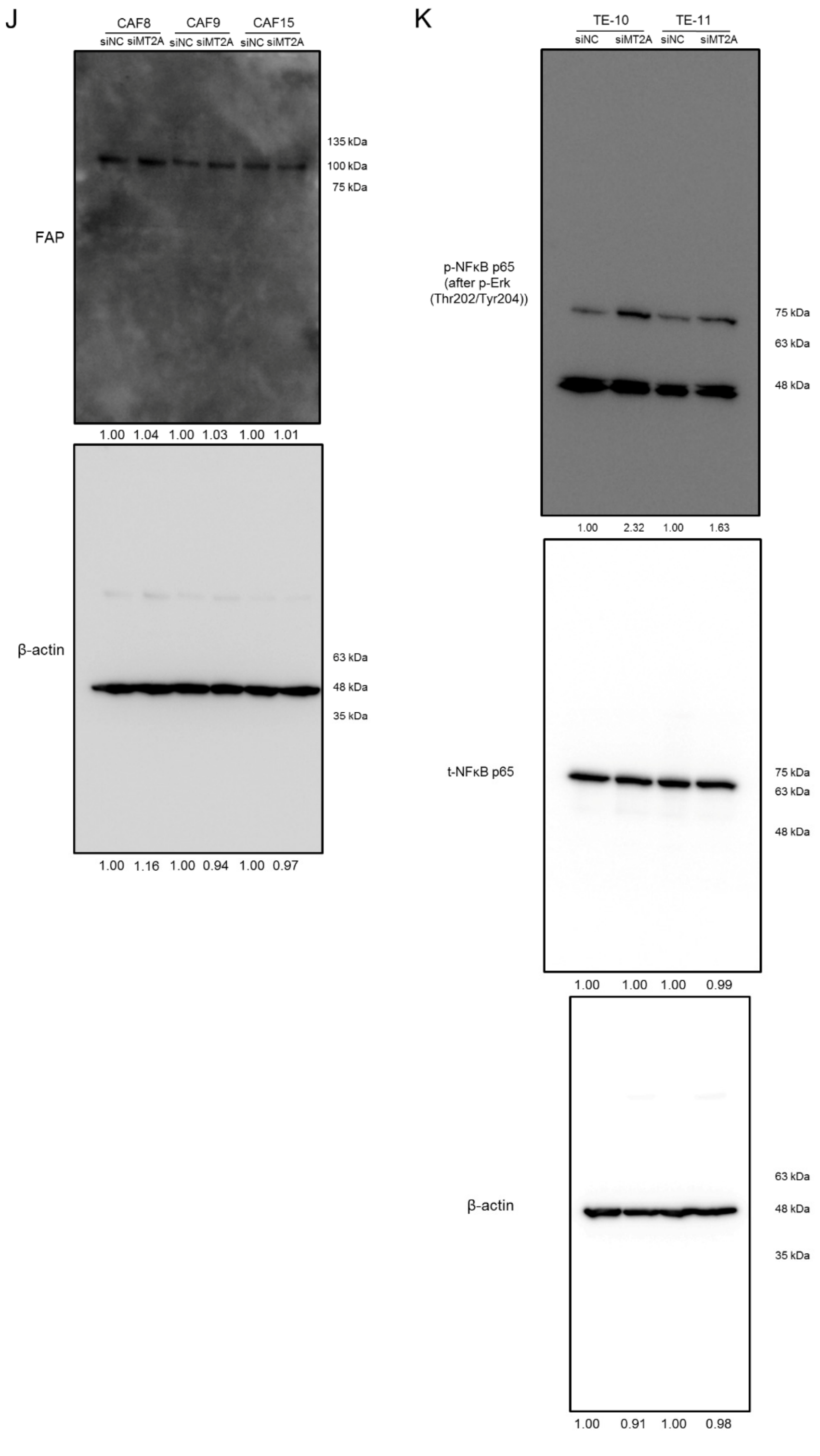

Figure S8. Uncropped the Western blotting images for Figures 1B, 1D, 2B, 2F, 2I, 5B, 5D, 5F, S1B, S3B, and S6, corresponding to Figures S8A–K, respectively. The Western blotting membrane was cut and used for immunoblotting several times to identify other bands. Because prestained markers of three colors, that do not contain chemiluminescent substances were used, the markers cannot be visualized in the raw data presented.

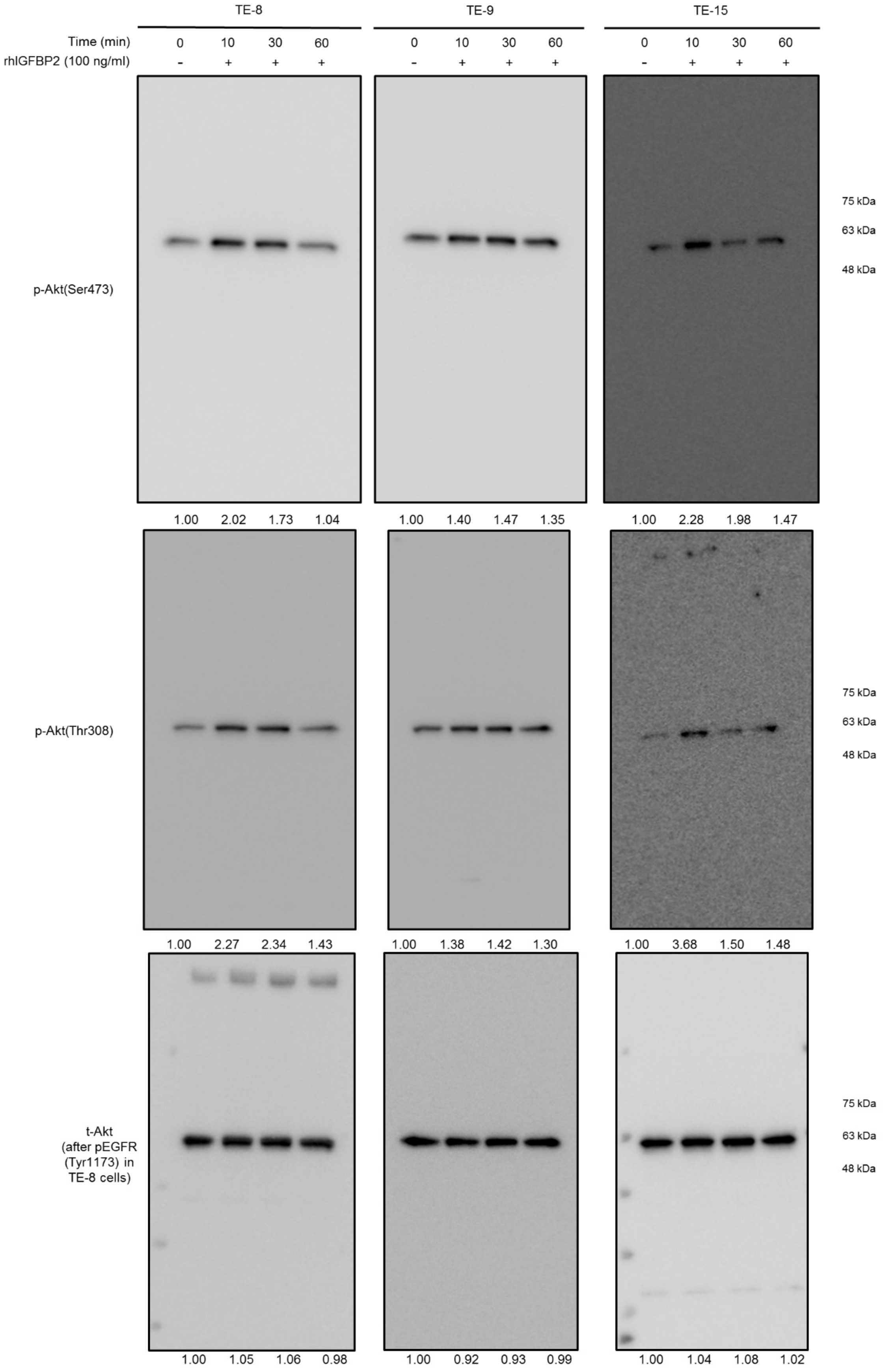
TE-8
TE-9
TE-15
Time (min)
0 10 30 60
rhIGFBP2 (100 ng/ml)
- + + +
p-Akt(Ser473)
75 kDa
63 kDa
48 kDa
1.00 2.02 1.73 1.04
1.00 1.40 1.47 1.35
1.00 2.28 1.98 1.47
p-Akt(Thr308)
1.00 2.27 2.34 1.43
1.00 1.38 1.42 1.30
1.00 3.68 1.50 1.48
t-Akt
(after pEGFR
(Tyr1173) in
TE-8 cells)
1.00 1.05 1.06 0.98
1.00 0.92 0.93 0.99
1.00 1.04 1.08 1.02

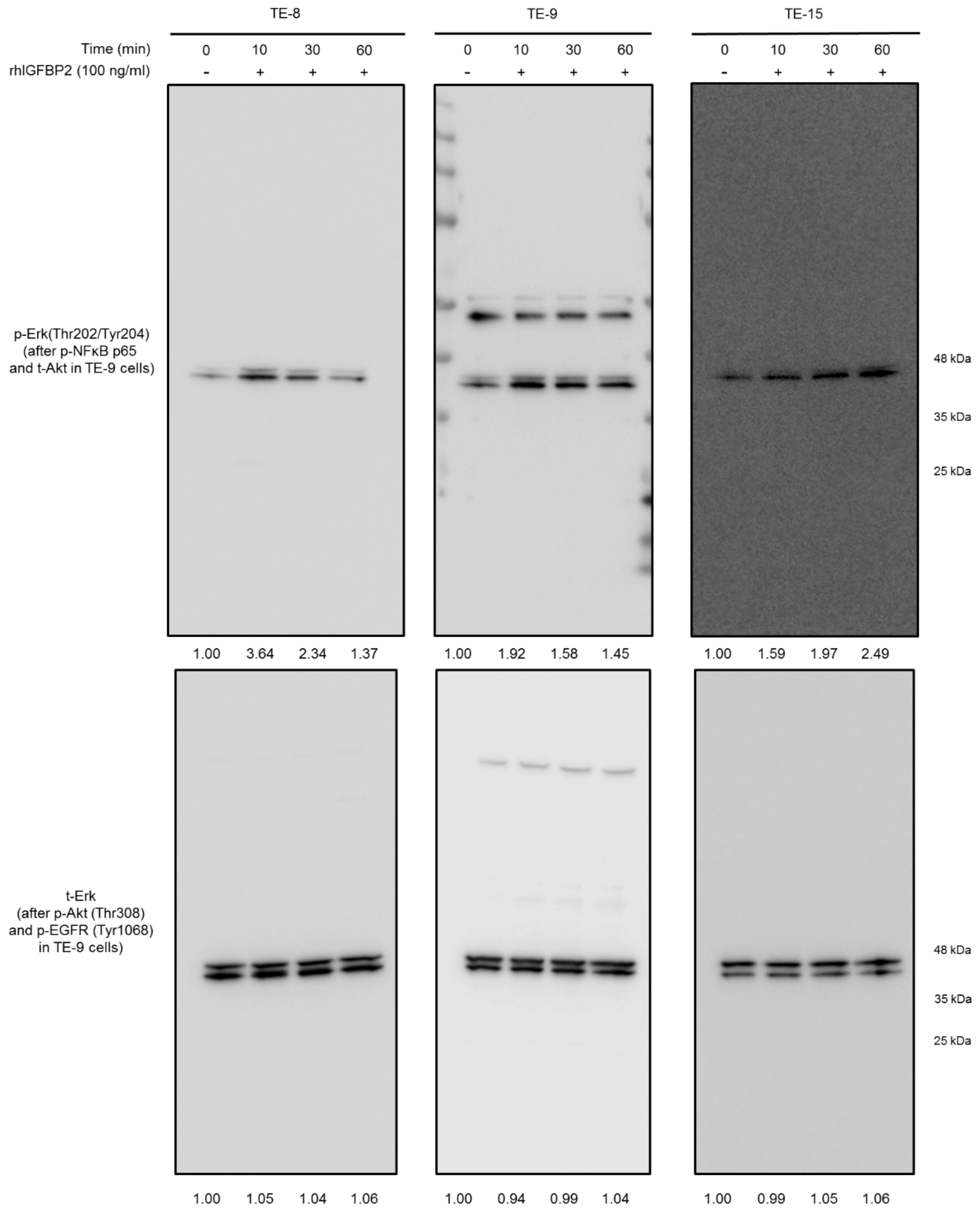
TE-8
TE-9
TE-15
Time (min)
0 10 30 60
rhIGFBP2 (100 ng/ml)
- + + +
p-Erk(Thr202/Tyr204)
(after p-NFκB p65
and t-Akt in TE-9 cells)
48 kDa
35 kDa
25 kDa
1.00 3.64 2.34 1.37
1.00 1.92 1.58 1.45
1.00 1.59 1.97 2.49
t-Erk
(after p-Akt (Thr308)
and p-EGFR (Tyr1068)
in TE-9 cells)
1.00 1.05 1.04 1.06
1.00 0.94 0.99 1.04
1.00 0.99 1.05 1.06

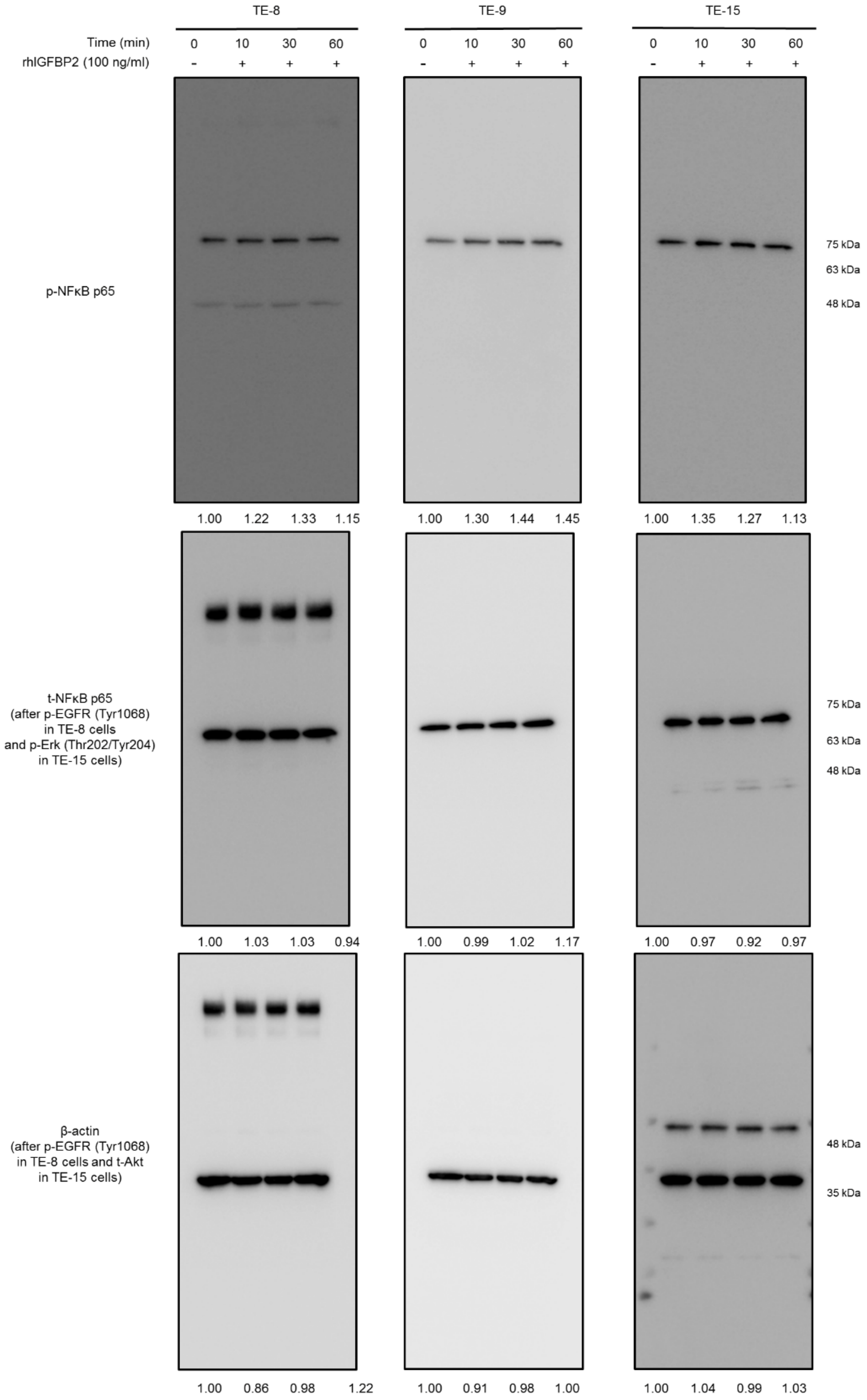

Figure S9. Uncropped the Western blotting images for Figure 4C. The Western blotting membranes were cut and used several times to identify other bands. Because prestained markers of three colors, that do not contain chemiluminescent substances were used, the markers cannot be visualized in the raw data presented.
